# Bioaccumulation and ecotoxicity of carbon nanotubes

**DOI:** 10.1186/1752-153X-7-154

**Published:** 2013-09-13

**Authors:** Petra Jackson, Nicklas Raun Jacobsen, Anders Baun, Renie Birkedal, Dana Kühnel, Keld Alstrup Jensen, Ulla Vogel, Håkan Wallin

**Affiliations:** 1 National Research Centre for the Working Environment, Lersø Parkallé 105, Copenhagen Ø, DK-2100, Denmark; 2 Department of Micro- and Nanotechnology, Technical University of Denmark, Kgs. Lyngby, DK-2800, Denmark; 3 Department of Environmental Engineering, Technical University of Denmark, Building 115, Kgs. Lyngby, DK-2800, Denmark; 4 Institute of Public Health, Copenhagen University, Øster Farimagsgade 5, Copenhagen K, DK-1014, Denmark; 5 Helmholtz-Centre for Environmental Research – UFZ, Permoser Str. 15, Leipzig, D-04318, Germany

**Keywords:** Carbon nanotubes (CNT), Single-walled carbon nanotubes (SWCNT), Double-walled carbon nanotubes (DWCNT), Multi-walled carbon nanotubes (MWCNT), Sorption, Bioaccumulation, Bacterial toxicity, Aquatic toxicity, Terrestrial toxicity, Hazard identification

## Abstract

Carbon nanotubes (CNT) have numerous industrial applications and may be released to the environment. In the aquatic environment, pristine or functionalized CNT have different dispersion behavior, potentially leading to different risks of exposure along the water column. Data included in this review indicate that CNT do not cross biological barriers readily. When internalized, only a minimal fraction of CNT translocate into organism body compartments. The reported CNT toxicity depends on exposure conditions, model organism, CNT-type, dispersion state and concentration. In the ecotoxicological tests, the aquatic organisms were generally found to be more sensitive than terrestrial organisms. Invertebrates were more sensitive than vertebrates. Single-walled CNT were found to be more toxic than double-/multi-walled CNT. Generally, the effect concentrations documented in literature were above current modeled average environmental concentrations. Measurement data are needed for estimation of environmental no-effect concentrations. Future studies with benchmark materials are needed to generate comparable results. Studies have to include better characterization of the starting materials, of the dispersions and of the biological fate, to obtain better knowledge of the exposure/effect relationships.

## Review

### Introduction

Release of carbon nanotubes (CNT) into the environment will rise with their increased production and widespread application in industrial and consumer products. Exposure and effect data are necessary for understanding the potential hazards posed by these new materials. Several scientific reviews have assessed the sources, behavior, fate, and the mechanisms of toxicity of nanomaterials in general (exemplified by specific nanomaterials) [1-15]. Most of these reviews commonly conclude that more research is needed in the field of nano-ecotoxicology and future studies have to include better particle and exposure characterization. Furthermore, it is often concluded that for the time being a risk assessment of nanomaterials can only be sensibly carried out on a case-by-case basis [16].

For CNT, a few scientific studies relevant for risk assessment have been published in recent years. While human toxicological assessment is well on the way [17,18], gaps still exist on the environmental hazard identification and effects/exposure assessment of CNT. However, experience with nanomaterials in ecotoxicological laboratories is improving and recommendations for systematic and comparable evaluations are emerging [19-21].

Carbon nanotubes are a heterogeneous group of nanomaterials and the industrial production and the number of applications is increasing rapidly. Numerous scientific papers describe their technical properties and applications [22-24]. Original studies on CNT environmental behavior, fate and ecotoxicity have been published in recent years, which is the topic of the current review. We summarize the most recent knowledge presented in peer-reviewed scientific literature with the focus on: a) CNT environmental fate in relation to interactions with other pollutants; b) CNT biological fate in living organisms; c) CNT effects on living organisms; d) including environmental hazard identification recommendations based on the presented literature.

The carbon nanotube toxicity may be influenced by a number of factors such as by the surface area, surface chemistry, functional groups, coatings, charge and aggregation, chemistry and solubility, shape, photochemistry, preparation method; as reviewed by [25]. Thus, the presence of contaminants retained during synthesis, the deliberate introduction of chemical groups during functionalization, or the presence of defects, may alter CNT toxicity. In the current review, all major types of CNT are included, and an integrated overview of modified toxicity by surface changes (both during industrial production and in the environmental media) is given.

## Methods

The following databases were searched for scientific literature with last search April 22, 2013: PubMed, Toxnet/Toxline, Scopus, SCI, Elsevier Science Direct, Google Scholar and Web of Knowledge. The search phrases were: ‘carbon nanotubes toxicity’ , ‘carbon nanotube toxicity’ , ‘carbon nanoparticles ecotoxicity’ , and ‘carbon nanotubes ecotoxicity’. Abstracts of all articles found were read and articles matching the scope of this review were selected. The number of articles found by the search databases is presented in Table 1. Overall one hundred and fifty four articles are used in the review. The particle and exposure characterization in all toxicological articles was screened, to evaluate the quality of the presented data and the validity of the hazard assessment in the review presented in Table 2. An overview with description and results of uptake and bioaccumulation studies, and ecotoxicity studies is supplied as a Additional file 1: Table S1.

**Table 1:** Search results for selection of articles used in the current review

**Search phrases:**	**Carbon nanotubes toxicity**	**Carbon nanotube toxicity**	**Carbon nanoparticles ecotoxicity**	**Carbon nanotubes ecotoxicity**
**PubMed**	779	779	17	17
**Toxnet/Toxline**	725	792	14	15
**SCI**	1850	1850	67	63
**Google scholar**	43200	23500	2490	1610
**ISI**	2611	2611	69	64

Last search 22. April 2013. Overall one hundred and fifty four references are used in the review.

**Table 2:** A status of CNT physicochemical characterization in the presented original articles

**YEAR**	**2004**	**2006**	**2007**	**2008**	**2009**	**2010**	**2011**	**2012**	2013*
**Total article number:**	1	2	7	12	17	14	14	9	1
**Manufacturer information only:**	0	0	0	2	4	2	2	1	0
**CNT quality: Raman, IR, NMR**	1	2	2	6	5	6	5	2	1
**CNT diameter, length &form: TEM, SEM, AFM**	1	2	5	9	10	11	12	7	1
**Elemental analysis:TGA, XRD, ICP-MS**	0	2	4	7	6	7	7	5	0
**Surface area: BET**	0	0	1	3	3	2	2	2	1
**Dispersion state in stock: DLS**	0	0	0	1	4	2	6	3	0

Used abbreviations: Raman spectroscopy (Raman), Infrared spectroscopy (IR), Nuclear magnetic resonance (NMR), Transmission electron microscopy (TEM), scanning electron microscopy (SEM), atomic force microscopy (AFM), Thermogravimetric Analysis (TGA), X-ray diffraction (XRD), Inductively coupled plasma mass spectrometry (ICP-MS), Adsorption of gas molecules on a surface (BET), Dynamic light scattering (DLS).

*Last search 22. April 2013.

## Carbon nanotube types, use and environmental release

**Figure 1:**
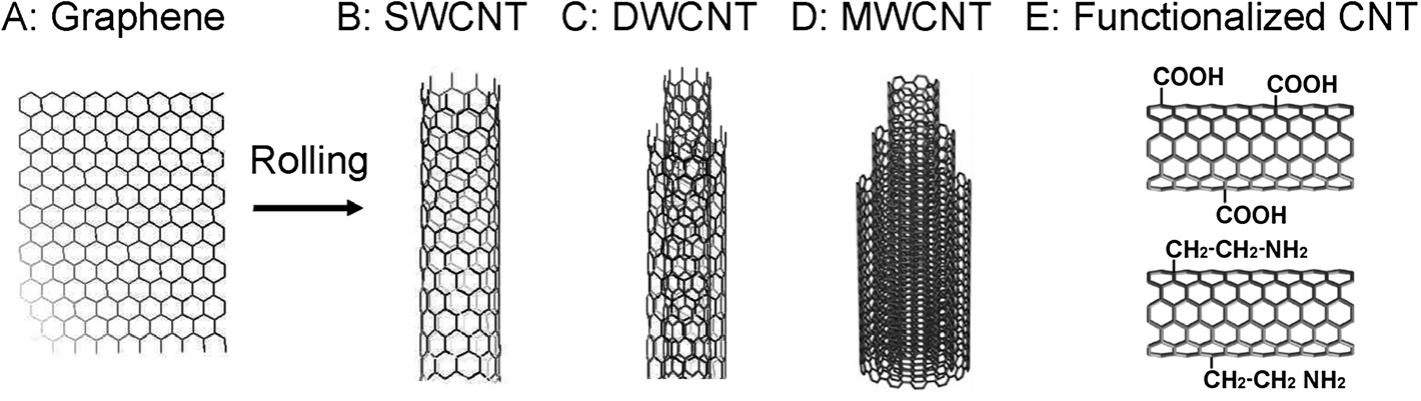
**Carbon nanotubes (CNT).** CNT are thin and long hollow fiber-like nanomaterials composed of a single, double or multiple layers of rolled graphene. The names are derived from the number of cyliders, known as single-walled carbon nanotubes (SWCNT), double-walled carbon nanotubes (DWCNT) or multi-walled carbon nanotubes (MWCNT). Surface modification by adding functional groups to the CNT surface (functionalization) is designed to change the surface properties and e.g. change CNT dispersion in the polar solvent (water). Presented is carboxylation and amine functionalization.

The first synthesized CNT [26], comprise a large group of thin (nanometers) and long (up to micrometers) hollow fiber-like nanomaterials. Several different types of nanotubes are produced. The current typical industrial types are: 1) CNT composed of a single cylinder known as single-walled carbon nanotubes (SWCNT); 2) CNT composed of two layers known as double-walled carbon nanotubes (DWCNT); and 3) CNT composed of multiple layers known as multi-walled carbon nanotubes (MWCNT) (Figure 1). Carbon nanotubes possess different physical and chemical characteristics (e.g., length to diameter ratios, atomic configuration, impurities, defects, and functionalization), and properties (e.g., conductivity, tensile strength, flexibility, and chemical reactivity) [23,27]. Most importantly for ecological hazard assessment, impurities may contain different carbon allotropes (e.g., graphite, soot, amorphous carbon and different CNT types) and several different transition and heavy metal catalyst nanoparticles (typically Fe, Ni, Co Au, and Pb as well as Al as catalyst substrate). These metal impurities may be either associated material or embedded metal or metal oxide particles in the CNT side walls and tube viods.

In the environment, CNT have been found in: 1) a natural form, as those in 10,000-year-old ice cores from Greenland [28]; 2) as incidentally generated, as CNT found in outdoor and indoor soot [29,30]; and 3) as engineered for many industrial applications [22]. Industrial production is expected to be the major source of CNT pollution in the future. Carbon nanotubes can enable several new materials and products, improve product performances, product lifetimes, energy savings etc. [22]. New applications range from reinforced composites, conductive materials, hydrogen storage media, drug delivery vessels, sensors and sorbents. Consequently, CNT are on the product list of several companies, some of which have high-tonnage production capacities. Still, limited knowledge exists on the actual and potential production volume as well as applications where CNT may be used. The estimated world-wide production is increasing rapidly and the production capacity is now exceeding several thousand tons per year [22]. In the future, CNT are expected to be used in drug delivery or in a broad range of environmental applications, such as sorbents, filters, antimicrobial agents, environmental sensors, renewable energy technologies, and pollution prevention strategies [31]. While CNT have great potential to contribute to environmental protection, more widespread use and higher volumes will inevitably contribute to the unwanted release into environment.

Carbon nanotubes may enter the environment directly during unintentional release during use and consumption of CNT containing goods or as a waste from sewage treatment plants, waste incineration plants and landfills [32]. Carbon nanotubes may be released intentionally in the future, as they are been explored for remediation and water cleaning purposes [33-38]. Based on a preliminary product life cycle analysis, CNT were characterized as ‘rather safe for the environment’ [39], because hazardous effects defined in [40] are not expected at current predicted exposure concentrations modeled by [41-43]. Moreover, CNT may be removed during waste incineration since they have been found to be completely destroyed at temperatures between 600-850°C [44,45], assuming proper burning. However, the currently predicted low average environmental concentrations will slowly rise due to increased CNT production and use [41-43]. Currently annual demand of CNT is estimated to increase from 3700–4100 tons to 5300–5720 in 2015 and finally 10500–12000 tons in 2020 [46]. The fate and impact of CNT in the environmental compartments will be affected by altering their surface properties. Several authors recently addressed in detail CNT environmental fate, including life cycle analysis [32,47-49]. Therefore this topic will not be discussed in detail in this review.

## Interactions with environmental media, organisms, and pollutants

Carbon nanotubes are difficult to disperse in water and polar matrices. Many commercially available CNT are therefore functionalized before final use. Typically the hydrophobic surface is oxidized or otherwise modified for better dispersion in polar solvents (including water) and to prevent agglomeration in the composite matrices. Additionally, dispersants can be added to the test media to reduce CNT agglomeration [50,51]. Similarly in the environment, natural coatings by e.g. organic matter will increase the pristine CNT dispersability in aquatic solutions by covering the hydrophobic surface. This reduces CNT agglomeration, prolongs residence time in the water column, increases CNT mobility and thus intensifies risk of exposure and toxicity [52-57]. Depending on length, diameter, entanglement, surface modification and environmental conditions, CNT may have a very different behavior in natural conditions and thus environmental fate.

Carbon nanotube stability in the aquatic environment may be influenced by water quality. Zhang et al. [56,58] reported that MWCNT stirred directly into test media aggregated and adsorbed to solids in hard and saline water, while they stayed stable in deionized water. Thus in hard or sea water the mobility of MWCNT will be low. In soil under saturated flow conditions, carboxylated COOH-SWCNT did not exhibit substantial transport and infiltration in soils because of an effective retention by the soil matrix [59]. Surface coatings may be activated after environmental release and change CNT toxicity. While pristine SWCNT dispersed with dispersant were not photoactive, functionalized (COOH- or PEG-) SWCNT produced reactive oxygen species when irradiated by sun light [60,61]. Thus, potential toxicity of CNT in the aquatic environment may increase by functionalization and sunlight.

Organisms can directly modify dispersion of CNT. MWCNT ingested by protozoan cells were excreted as granules in micron size and sedimented [62]. Transfer via the *Daphnia magna* digestive system removed lipid coating used for increasing SWCNT water solubility and subsequently made CNT less water soluble and more prone to sedimentation [63], a behavior also observed in by [53]. Also otherwise stable MWCNT destabilized, agglomerated and sedimented in the presence of *Xenopus leavis* larvae and their food [64].

The large specific surface area may accommodate pollutant adhesion and thus influence CNT toxicity in itself and/or toxicity of co-pollutants [53]. The surface area, a function of outer diameter and pore volume, may determine the adsorption capacity. Sorption effects of CNT to different pollutants present in the environment has been studied by several authors [33-38,53,65-72].

The adsorption of Ibuprofen and Triclosan to SWCNT, MWCNT and oxidized O-MWCNT was analyzed as models of environmentally relevant contaminants [34]. Surface chemistry as well as aqueous solution chemistry influenced the adsorption to the studied CNT, depending on: 1) the specific surface area available (SWCNT >MWCNT>O-MWCNT); 2) solution pH in relation to CNT pKa value, increased sorption at pH below pKa; 3) ionic strength in the solution (saline solution facilitated CNT agglomeration, adsorbing Ibuprofen while competing with Triclosan; 4) presence of organic matter (fulvic acid) reduced adsorption due to competition [34]. Norfloxacin, an antibiotic contaminant released to the environment was studied for sorption to MWCNT with three surface functionalisations (graphitization, carboxylation, hydroxylation). The purpose was to find an efficient sorbent for this contaminant [70]. Even though MWCNT were less efficient sorbents compared to activated carbon, they were able to absorb Norfloxacin and this sorption was influenced by chemical surface modification.

Nanomaterials are suspected to enhance the transport of hydrophobic organic contaminants (HOC) in porous media if they are: 1) present in high concentrations; 2) stable in media; 3) and have high sorption affinity [36]. Carbon nanotubes specifically have a great sorption capacity. Therefore, the presence of CNT in the environment may affect the bioavailability of HOC. SWCNT adsorb the model HOC, phenanthrene, similarly to activated carbon [71]. The 7d adsorption capacity of aromatic hydrocarbons to CNT with different characteristics was investigated in mixtures of distilled and deionized water added 1, 10, 100 and 1000 mg/L phenantrene pre-dissolved in methanol [33]. The results showed that the SWCNT had a greater adsorption capacity than three different MWCNT. At concentrations 1–10 mg/L, the phenantrene adsorption appeared to be linked to the tube diameter (curvature), but at higher concentrations the adsorption capacity was increasingly controlled by the specific surface area [33]. In the same study the smaller HOC molecule, naphthalene, was adsorbed less efficiently and the CNT surface area affected the process insignificantly. The adsorption capacity of various polycyclic aromatic hydrocarbons (PAH) to low concentrations of MWCNT was linear and directly related to the total surface area [66]. Thus HOC adsorption capacity may be influenced by CNT surface area and by surface treatment. Oxidized MWCNT had reduced adsorption capacity compared to the pristine product in a linear relationship (10% increase in O_2_ content reduced sorption by 70%) [65]. Pristine MWCNT adsorbed more than natural char, but less than granulated activated carbon [65]. Similarly, MWCNT coated with dissolved organic matter (DOM) showed reduced HOC adsorption, compared to non-coated MWCNT [37]. Alike, SWCNT dispersed in dispersant cetylpyridinium chloride had reduced adsorption to naphthalene [38]. The dispersant occupied available SWCNT surface, thereby reducing the surface area by 8-fold. Consequently, surface treatment of CNT can alter CNT chemical characteristics, reduce CNT surface area, ultimately reduce the ability to adsorb organic contaminants from water, and hence also changes the interaction with organisms.

CNT presence can further influence the biological degradability and bioavailability of pollutants [35]. SWCNT reduced bacterial degradability of phenanthrene more efficiently than biochar and charcoal. The effect was reduced by the presence of DOM [35], due to reduced surface area. The presence of CNT may also influence bioaccumulation of environmental contaminants. The uptake of HOC from aquatic sediments by two infaunal deposit feeders was compared in presence and absence of SWCNT [53]. Addition of SWCNT to aquatic media significantly reduced bioaccumulation of HOC in deposit/suspension feeding polychaete *Streblospio benedicti*, while bioaccumulation in deposit-feeding meiobentic copepod *Amphiascus tenuiremis* was less affected [53]. Addition of MWCNT to sediment spiked with HOC or perfluorochemicals (PFC) reduced bioaccumulation of these chemicals in benthic developing larvae of *Chironomus plumosus*[73,74]. Both chemical types were removed most efficiently from the aqueous phase when MWCNT concentration was below 1.5% dry sediment weight. At higher concentrations the bioaccumulation increased, probably because larvae ingested the MWCNT-associated pollutants [73,74]. In soil, the presence of SWCNT and MWCNT in high concentrations (3 g/kg) decreased pyrene bioaccumulation in a terrestrial polychaete *Eisenia foetida*, because CNT decreased uptake and increased PAH elimination [69]. At the same time, MWCNT were reported to pierce plant (wheat) roots and facilitate phenanthrene transport into the cells [75]. Thus, both in the aquatic and terrestrial environment, CNT would alter the adverse effects of pre-existing HOC, because adsorption to the CNT would influence the bioavailability and possibly biodegradation. In addition, mechanical damage to tissues induced by the fibre form may facilitate HOC transport and bioaccumulation.

The presence of CNT may also change the environmental fate of metals. The bioavailability and acute toxicity of copper (Cu) (to *D. magna* and ROS reactive oxygen species production) was increased in the presence of MWCNT dispersed in natural organic matter (NOM) [67]. Similarly, surface lysophosphatidylcholine modified SWCNT additively enhanced bioavailability, uptake and toxicity of Cu in the aquatic environment [68]. Thus, CNT can bind to NOM in competition with metal ions and this may increase their bioavailability and toxicity. Interestingly, the presence of Cu ions may increase the adsorption of aromatic compounds to surface modified SWCNT [76]. Copper ions may form complexes with both SWCNT functional groups and phenolic and amino molecules in solution, hence acting as bridging agents between CNT and organic contaminants.

CNT sorbent properties will be explored in the future for removal of chemicals in polluted environments. However, it still needs to be explored how the CNT toxicity would be affected during interaction between accidentally released CNT and already present pollutants.

## Uptake and bioaccumulation

Successful CNT uptake, translocation and retention in the exposed organism are important prerequisites for bioaccumulation in the body. The main routes of entry into the organism are the body surface, relevant for animals and plants, as well as digestive and respiratory system. The studies in the following section explore bioaccumulation through various animal and plant models, with the focus on uptake and excretion.

Fresh water protozoans *Tetrahymena thermophila* and *Stylonychia mutilus* ingested and excreted SWCNT and MWCNT [77]. Protozoan *T. thermophila* ingested CNT and bacterial food without any discrimination. Consequently, CNT impaired bacterivory (ingestion of bacteria by phagocytosis), and impaired *T. thermophila* in regulation of bacterial growth [77]. MWCNT were transferred from *S. mutilus* parental cell to the two daughter cells during cell division [62]. Thus, the ingested CNT may affect protozoan food intake, and could be transferred between generations and move up the food chain.

The fresh water flea *D. magna,* a planktonic crustacean, is a model organism commonly used in aquatic ecotoxicology studies. SWCNT and MWCNT with different lengths and surface treatments have been tested in acute toxicity tests as well as in shorter and longer term bioaccumulation/elimination studies [52,63,78-80]. Elimination was limited or not possible in absence of algal food [52,78,79]. In the presence of food, CNT aggregated in *D. magna* gut, affected food processing, which likely contributed to the toxicity. However, CNT were not able to cross the gut lumen [52,78,79]. The absence of food in the media prolonged the time-to-elimination of MWCNT by almost a day, while in presence of food the elimination took only few hours [52]. Furthermore, the presence of NOM in media did not influence the time-to-elimination [52]. Another fresh water flea *Ceriodaphnia dubia*, also ingested and defecated MWCNT despite of their different lengths, however sample preparation had significant effect on CNT retention without effect on toxicity (retention sonication>ozone treatment; while toxicity ozone>sonication>stirring) [81]. Similarly to *D. magna*, *C. dubia* eliminated CNT only in the presence of food [57]. The difficulty to clear the large CNT agglomerates from the gut likely caused *C. dubia* immobilization and mortality [57]. An estuarine crustacean, *Tigriopus japonicus,* ingested and excreted DWCNT without further internalization [82]. Analysis of *T. japonicus* clearly indicated that despite ingestion, DWCNT were not present in the cuticle or cuticle cells [82]. Thus, crustaceans can be expected to ingest CNT regardless of type and behavior in environmental media. The ingested CNT may interfere with crustacean food intake and movement, which may induce toxicity and disturb their ecosystem function. When crustaceans are ingested by higher organisms CNT may move up in the food chain.

Sediment living meiobenthic crustacean *A. tenuiremis* (a free-burrowing copepod) and polychaete *S. benedicti* (a tube-dwelling worm), were observed to ingest and subsequently eliminate SWCNT without any sign of bioaccumulation [53,83]. It was observed that *A. tenuiremis* ingested SWCNT as aggregated clusters with algae, and clusters were egested as smaller tightly packed clusters [83]. Similarly, marine infaunal lugworm *Arenicola marina* did not bioaccumulate SWCNT into tissues, the CNT either remained in the sediment or passed through the gut and were excreted [84]. An infaunal lugworm *Lumbriculus variegatus* exposed in longer term bioaccumulation and elimination studies did not absorb SWCNT or MWCNT via dermis or gut tissue after ingestion, and CNT were eliminated [85,86]. A similar behavior was observed for the soil dwelling earthworm *Eisenia foetida*[69,86-88]. The studies with sediment and soil living organisms suggest that both SWCNT and MWCNT, irrespectively of the surface treatment and environmental contaminants present, do not translocate outside the digestive system, even though uptake into the gut and elimination with feces were observed.

In aquatic vertebrates, bioaccumulation was assessed during early developmental stages. Zebrafish embryos were specifically assessed for bioaccumulation of SWCNT and MWCNT [89,90]. The chorion acted as a strong protective barrier and prevented passage, even though SWCNT adhered directly to it [89]. Fluorescent-labelled MWCNT were injected into one-cell-stage zebrafish embryos [90]. CNT allocated to blastoderm cells of the embryos through proliferation and were excluted from the yolk cell. When introduced into the circulation system, MWCNT moved easily in the compartments and were finally cleaned out 96 h after injection [90]. Thus regardless of CNT form and exposure type, CNT did not enter the embryo or were cleared early after exposure without affecting development. The amphibian species *Xenopus laevis* larvae were exposed to MWCNT or DWCNT for 12d [91-93]. Both types of CNT suspended in water were detected in the lumen of the intestine, but not in intestinal cells nor in circulating blood of exposed the amphibian larvae, suggesting that CNT do not cross the intestinal cells. Observed toxicity was likely induced by physical blockage of gills and digestive tract [91-93]. Thus at low doses CNT did not cross readily into the embryo, and if internalized in blood or digestive system, they tended to clear out of the body without affecting development. However, the CNT presence on or in the body may induce toxicity.

Little information is available on the biodistribution in terrestrial organisms. As a model terrestrial organism, the common fruit fly *Drosophila melanogaster*, was fed dry yeast which was spiked with SWCNT or MWCNT [94]. Drosophila ingested SWCNT and a small fraction translocated into the hemolymph, to the brain and to a lesser extend to other tissues. Majority of SWCNT were excreted [94]. At higher dose, larval ingestion lead to systemic SWCNT and MWCNT uptake and tissue integration [95]. Currently, no studies are available with wild terrestrial species from higher trophic levels e.g. birds and rodents. It could be expected that ingestion of lower organism, drinking water or soil containing CNT would be the most prevalent route of exposure. Two studies with laboratory rodents assessed distribution following ingestion of CNT. Three hours after oral administration of short hydroxylated SWCNT, they were detected at high concentrations in stomach, kidney, lungs, bone, and low concentrations were found in brain, heart and muscle [96]. Deng and co-workers demonstrated that 12 h after ingestion of radioactively labeled taurine fuctionalized MWCNT, 75% of CNT were excreted in feces. No labeling was detected in blood, suggesting that MWCNT were not absorbed from gastrointestinal tract in detectable concentrations [97], lymphatic system and liver were not analyzed. The studies suggest that depending on type, functionalization and behavior in the media, ingested CNT may cross from the digestive system into other body compartments and organs, however translocation is expected to be low and CNT would be excreted again.

The biodistribution of CNT in plants has been studied in several models. In a root elongation study, cucumber seedlings (*Cucumis sativus*) were exposed for 48 h to SWCNT that were non-functionalized or functionalized with poly-3-aminobenzenesulfonic acid. Both CNT were present on the root surfaces, but no visible uptake was observed [98]. As a first long-term study [99], rice seeds *Oryza sativa* were pretreated with MWCNT, suspended and sonicated in NOM solution at concentrations up to 800 mg/L, and plants were allowed to grow for 6m. A few aggregates were observed in the vascular system and almost none in the plant tissues [99]. In contrast, when mustard and tomato seeds were germinated in the presence of pristine or oxidized MWCNT, CNT penetrated the seed coat and the root tissue [100,101]. Also wheat roots grown in the presence of MWCNT were pierced by the CNT, though CNT did not fully enter in the cells [75]. Moreover tomato plants germinated and grown in medium with COOH-MWCNT were able to take up CNT and biodistribute them into roots, leaves and fruits [102]. In a recent study uptake of ^14^C-MWCNT was quantified in wheat *Triticum aestivum* and rapeseed *Brasica napus*[103]. Results demonstrated that less than 0.005‰ of the applied dose was taken up by roots and leaves. CNT accumulated in newly developed leaves and stayed in peripheral areas [103]. Thus, even though uptake of CNT is possible, it is at limited concentrations. However, the CNT/plant interaction may affect the plant physiology. Piercing of seeds, roots and plant transport was reported to induce beneficial (at low doses), none or negative effects (at high doses) [101]. Especially beneficial effects are interesting, increased water transport is suggested to cause the induced germination or growth [100-102,104]. An induced uptake of pollutants or nutrients by the same route may though also be possible [75]. More studies are needed to understand the CNT biodistribution and possible bioaccumulation in plants. The focus on plants under natural soil conditions is especially important, since the presence of soil microorganisms or organic matter may influence CNT uptake.

The presented bioaccumulation studies provide evidence that CNT are ingested by invertebrate and vertebrate organisms and are subsequently excreted [52,53,55,62-64,69,77-88,91-94,97]. Thus bioaccumulation of CNT in the individual organism may be minimal. CNT were able to penetrate into plant tissues [75,100-102]. Therefore, organisms containing CNT may become source of entry of CNT into the food chain when ingested by larger animals, potentially leading to biomagnification.

## Effects in living organisms

### Effects on microorganisms

The proposed mechanisms of antimicrobial action of CNT are: 1) membrane integrity disruption by a strong electrostatic interaction between bacteria and CNT; and/or oxidation of the membrane; or by membrane puncture; 2) reactive oxygen species may directly interact with organelles or indirectly induce DNA damage or protein inactivation leading to cell death or apoptosis in eukaryotes; 3) impurity toxicity; 4) bacterial agglomeration [95,105-111].

Carbon nanotubes may cause damage to planktonic microorganisms, as well as to microorganisms present in soil and on solid surfaces. Planktonic microorganisms play a key role in nutrient recycling affecting productivity in surface waters, moreover nanomaterial-bacteria agglomerates may shadow aquatic plants, reduce photosynthesis and plant biomass available for aquatic animals [12]. Nanomaterial induced toxicity to microbes in the soil may affect phyto-production, organic matter breakdown, nutrient recycling, groundwater purification, and soil creation, stability and infiltration capacity [12]. Increased use of nanomaterials, including CNT, increases the likelihood that microorganisms used in industrial processes will also be affected, which may be a problem for e.g. wastewater treatment plants [106,107,112].

SWCNT possess powerful antimicrobial activity on both suspended and deposited bacteria, and affect the formation of bacterial films. The direct close contact between the SWCNT and bacteria is proposed to cause bacterial cell death [105]. Individually dispersed SWCNT were more toxic than agglomerates, due to increased efficiency in physical puncturing of bacterial membranes and degradation of bacterial cell integrity [113]. The degree of CNT-bacteria aggregation was influenced by CNT functionalization and length may modulate the toxic effect on the bacteria. Neutral or negatively charged SWCNT functionalized with OH- or COOH- aggregated more efficiently with bacteria and reduced bacteria viability, as compared to the positively charged SWCNT, functionalized with NH2- [110]. Similarly, longer SWCNT were observed to aggregate with bacteria inducing toxicity in a concentration and time dependent manner, while short SWCNT aggregated alone and therefore were less toxic [111]. Purity of SWCNT may also influence the bacterial toxicity. Higher metal content of SWCNT induced more bacterial toxicity compared to more pure SWCNT, toxicity was mediated by glutathione oxidation that occurred shortly after contact [108]. Moreover, higher ionic strength in suspensions, such as Phosphate Buffered Saline or Brain Heart Infusion broth, also reduced SWCNT toxicity, compared to low ionic strength suspensions (deionized water or saline) [110]. High ionic strength might reduce the intensity of the interactions between SWCNT and cells [110]. Coating by NOM reduced SWCNT toxicity, despite reduced number of aggregates [114], possibly by reducing SWCNT and cell interactions. In soil, SWCNT reduced enzyme activity and microbial biomass at concentration 300 mg/kg and higher [115]. Since SWCNT clearly induce bacterial death, surface coating with SWCNT would reduce biofilm formation both in natural and industrial environments [116].

MWCNT seem to be less toxic to bacteria as compared to SWCNT [110,114,117,118]. The reduced toxicity may be caused by less tight interactions between bacteria and MWCNT, due to the higher inherent rigidity and possibly smaller van der Waal’s forces at the MWCNT surface [110]. For the same reason, thin MWCNT with smaller diameter induce higher toxicity than the thicker ones [118]. When the effect of length of MWCNT was assessed, shorter MWCNT were more toxic to *Pseudomonas fluorescens* compared to long MWCNT [119]. Both lengths affected membrane structure integrity and DNA, likely by inducing reactive oxygen species increasing with dose for both MWCNT [119]. Toxicity of thin and short CNT was probably attributed to greater membrane interaction. When MWCNT are uncapped, debundled, short and dispersed in solution, the toxicity increased [120]. The purity of CNT has also been suggested to affect the toxicity. However, when comparing the toxicity between MWCNT in raw form (Fe as catalyst) and purified (heat-treated) in two bacterial strains, no difference in toxicity between the two forms of MWCNT was observed [121], Heating purification possibly has limited the ability to modificate the surface compared to acid treatment, thus preserves toxicity of the raw form [121]. However, both studied CNT were suspended in the presence of Gum Arabic (GA, 0.25 wt%), which may have modified their surface, affecting the toxicity. The MWCNT were toxic to a sensitive *Escherichia coli* strain while a pollutant resistant strain of *Cupriavidus metallidurans* was not affected [121]. In soil, MWCNT reduced enzyme activity and microbial biomass at concentration 5000 mg/kg [122]. The higher surface areas of SWCNT [115], compared to MWCNT [122], may affect the soil toxicity. Supporting this hypothesis, the LOEC for the two studies was 300 mg/kg and 5000 mg/kg, respectively [115].

One study evaluated the effects of MWCNT on fungal growth [123]. Entomopathogenic fungi *Paecilomyces fumosoroseus* conidia were incubated with 0.2 mg/L raw or carboxylated MWCNT for 1 h and up to 865 h. After incubation sporulation and mycelium growth on solid medium were recorded. Sporulation increased after shorter exposures and reducted after longer exposures for both types of CNT. Exposure had no significant effect on fungal growth and biomass production, other than reduction of biomass after exposure to raw MWCNT for 865 h [123]. Effects were likely induced by mechanical effects of CNT, as observed for bacteria.

In a complex natural system, with many reactive particles and large surfaces, interaction of nanoparticles with planktonic microorganisms will be a less common event. Bacteria in aquatic, subsurface and soil environments tend to attach to surfaces, thus biofilm communities may be a better model for bacterial toxicity, compared to planktonic cells [124]. Only few studies are available on the CNT effects in complex environmental samples [106,114-116], while many studies were performed with bacterial monocultures [105,108,110,111,113,117-121]. From those can be generalized that the CNT size and surface characteristics can influence microbial toxicity. Similarly, microbial toxicity depends on external environmental factors such as presence of NOM. Higher toxicity was observed for SWCNT that were well dispersed, negatively charged, and with higher metal content; compared to agglomerated, positively charged, and pure CNT. SWCNT were reported to be more toxic compared to MWCNT. Similarly, higher toxicity was observed for MWCNT that were thinner, shorter and de-bundled; compared to thicker, longer and tangled CNT. The observed toxic effects of CNT were related to improved ability to interact with the microbial wall. Surface functionalization, coating, or addition of dispersants increased or decreased CNT toxicity, depending on the character of the treatment.

### Effects on aquatic species

#### Effects on aquatic autotrophic and heterotrophic microorganisms

Similarly to bacteria, toxic effects of CNT in algae and unicellular protozoa are mostly driven by a direct contact with the surface. Algal growth can be inhibited by CNT shading and formation of algae-CNT agglomerates, as suggested in a study with two fresh water green algae *Chlorella vulgaris* and *Pseudokirchneriella subcapitata* exposed to pristine or oxidized CNT suspended in algal test medium by sonication [125]. After 96 h exposure in a well dispersed CNT solution the *C. vulgaris* growth was inhibited at Lowest Observed Exposure Concentration (LOEC) of 0.053 mg/L for both pristine and oxidized CNT, with Effect Concentrations 50% (EC50) of 1.8 and 2.5 mg/L, respectively. *P. subcapitata* had reduced growth after the same exposure time to the well dispersed pristine CNT at LOEC 5.5 mg/L (EC50 20 mg/L), thus it was less sensitive to the exposure [125]. In a longer exposure for 4 or 14d , *P. subcapitata* was exposed to well-dispersed SWCNT in the presence of the dispersant GA at concentrations 0.023% or 0.046% (v/v) [109]. After 4d exposure to SWCNT, algal growth was inhibited at LOEC 0.25 mg/L in the presence of 0.023% (v/v) GA, while the double concentration of GA reduced this effect with No Observed Effect Concentration (NOEC) 0.5 mg/L. In fact, a slight stimulatory effect was observed for this test group [109]. During 14d exposure, *P. subcapitata* recovered from the initial growth inhibition [109]. When green algae *C. vulgaris* was exposed for 96 h to MWCNT of diameter 10, 20–40, and 60–100 nm dispersed by sonication, growth was inhibited at EC50 41.0, 12.7, and 12.4 mg/L, respectively [126]. Under dark conditions, however, toxicity was lower with EC50 values of 62.2, 36.8 and 46.3 mg/L, respectively [126]. The contribution of metal catalyst impurities as well as adsorption of nutrients to the growth inhibition was found to be negligible; MWCNT toxicity toward *C. vulgaris* was mainly a combined effect of oxidative stress, agglomeration, physical interactions, and shading [126]. When marine diatom *Thalassiosira pseudonana* was exposed to DWCNT dispersed by sonication or stirring for 96 h, the sonicated DWCNT were more toxic than the stirred [82]. Algal growth was reduced at 96 h with LOEC 0.1 mg/L (EC50 1.86 mg/L) and LOEC 0.1 mg/L (EC50 22.7 mg/L) for the sonicated and stirred DWCNT, respectively [82]. In another study, a marine algae *Dunaliella tertiolecta* was exposed to carboxylated MWCNT in a 96 h algal bioassay [127]. A lag in the growth phase was observed starting at 5 mg/L with EC50 96 h growth at 0.8 mg/L, and oxidative stress and photosynthesis inhibition were reported at LOEC 10 mg/L. When the MWCNT suspension was filtered through 0.2 μm filters, all above observed effects disappeared [127]. In a chronic toxicity test, a unicellular ciliated protozoa *Tetrahymena thermophila* was exposed to oxidized SWCNT for 96 h [77]. An initial loss of mobility and cell death were observed at LOEC 1.6 mg/L leading to viability loss at LOEC 6.8 mg/L after 96 h exposure. Since the presence of the SWCNT also inhibited bacterivory with the LOEC 3.6 mg/L, exposure may disrupt the protozoan ecological role in regulation of bacterial populations [77]. Similarly for a unicellular protozoan *Stylonychia mytilus*, when exposed to functionalized MWCNT for 5d, cellular growth was inhibited at LOEC 1 mg/L starting at 24 h after exposure, with increased effects with time [62]. Surprisingly, low dose of MWCNT stimulated *S. mytilus* growth [128], supporting the paradigm observed in later field study [129].

Thus both fresh water or marine algae and unicellular protozoans are sensitive to the exposure to CNT, similarly to bacteria the toxicity is likely induced by direct contact between the cell and the CNT. It is still uncertain whether algae have the ability to recover from the initial CNT exposure, while protozoans respond negatively to the accumulative exposure over time. It is possible that observations are specific for type of CNT, dispersion media or exposed species. Similarly, it is uncertain if the well-dispersed compared to the agglomerated material induces greater toxicity. More comparative studies where a single factors are varied at a time are needed to address these questions.

#### Effects on pelagic and benthic invertebrates

Many studies are available addressing aquatic toxicity in fresh water, estuarine and marine invertebrates, living both in the water column as well as in the benthos. The majority of studies assess CNT effects on single species in laboratory settings. A single study addressed effects of MWCNT contamination in the sediment on a benthic macroinvertebrate community [129]. Natural sediment was spiked with concentrations of 0.002 to 2 g/kg (d/w) MWCNT and was returned to the original site for 3m. Benthic organisms and aquatic macrophites were identified to assess the effect of CNT pollution on invertebrate re-colonization. The numbers of individual taxa increased with increasing MWCNT concentration (especially macrophytes). Loss of biodiversity and effects on population level were not detected at the examined concentrations, which were assumed to be environmentally relevant [129]. This study is unique by showing the opposite trend to other laboratory studies. It is possible that the sub-toxic concentrations of CNT introduce a slight stimulatory effect by up-regulation of repair mechanisms, a paradigm observed for other pollutants discussed by [130].

Effects of CNT in the water column and on bentic organisms including decomposers, primary producers, primary and secondary consumers (e.g. bacteria, algae, crustacea) were studied in laboratory experiments [131]. The analyzed SWCNT were less toxic compared to other inorganic nano-powders, with toxicity LOEC 1–10 mg/L for algae and hydroid crustacean *Hydra attenuata*, while in all other assays toxicity was above 100 mg/L (NOEC) [131]. Toxicity was therefore species specific and possibly influenced by CNT availability.

The immobilization and mortality of *D. magna* in the presence of SWCNT have been studied with test durations 24, 48 and 96 h after CNT exposure. SWCNT (60% pure) re-suspended by shaking in water induced 48 h immobilization at EC50 1.3 mg/L and mortality at Lethal Concentration 50% (LC50) 2.4 mg/L [80]. A liposacharide coated SWCNT induced 48 h mortality at LC50 6.1 mg/L and 96 h mortality LC50 at 0.05 mg/L [68]. A lysophosphatidylchlorine solubilized SWCNT induced 20% mortality after 96 h with a LOEC of 10 mg/L [63] (LC50 ~2.5 mg/L specified in [67]). Depending on the length of exposure and type of SWCNT, the lowest effect concentration in the presented studies ranged from 2.4-6.1 mg/L for 48 h mortality and 0.05-2.5 mg/L for 96 h mortality. Consequently SWCNT were more toxic after longer exposure. Daphnia immobilization by SWCNT was tested only in one study, where 48 h immobilization occurred at EC50 1.3 mg/L [80]. This concentration was 50% lower to the lowest reported concentration that induced 48 h EC50 mortality [80]. Therefore, it can be expected that SWCNT would affect *Daphnia* populations at concentrations lower than presented in mortality studies.

The immobilization and mortality of *D. magna* was also studied in the presence of MWCNT. MWCNT re-suspended in NOM did not induce *D. magna* 48 h mortality even at 20 mg/L (NOEC), while prolonged exposure for 96 h induced mortality at LC50 2.5 mg/L [67]. MWCNT re-suspended in NOM for stabilization induced *D. magna* 96 h mortality at LC50 2–4 mg/L, depending on the NOM type, and reduced growth at LOEC 0.25 mg/L [52]. In another study, *D. magna* was exposed to MWCNT acid treated or MWCNT grafted with polyethylenimine (PEI) [79]. The two CNT induced immobilization with EC50 for 24 h exposure at ~25 mg/L and EC50 for 48 h exposure at 12.7 mg/L, or EC50 for 24 h exposure at ~17 mg/L and EC50 for 48 h exposure at ~9 mg/L, MWCNT acid treated or PEI grafted respectively. Increased toxicity due to PEI treatment was due to increased size of the surface coating, and not due to surface charge as otherwise expected [79]. MWCNT (98% pure) re-suspended by shaking in water induced 48 h immobilization at EC50 8.7 mg/L and mortality at LC50 22.8 mg/L [80]. Reproductive function (reaching three broods) of *D. magna* was evaluated by 21d exposure to MWCNT stabilized by NOM. At pH 7, 45% reduction in reproductive means was observed at LOEC 0.24 mg/L [132]. To summarize, different MWCNT induced *D. magna* mortality at concentrations above 20 mg/L for 48 h exposure and around 2 mg/L after 96 h exposure. Sub-toxic parameters such as growth or reproduction were affected at concentrations as low as 0.2 mg/L, similarly as observed for SWCNT. Immobilization was a less sensitive parameter, with effects ranging from 9 to 25 mg/L, depending on the particle type and exposure length. Comparing effects of SWCNT and MWCNT, the latter were less toxic to *D. magna*.

The fresh water flea *C. dubia* was exposed to MWCNT re-suspended in the presence of NOM in a 7d reproduction study [52]. No *C. dubia* mortality was observed up to 1 mg/L (NOEC), growth was affected at 0.2 mg/L (Pers. Comm. A.P. Roberts), and reproduction was reduced at LOEC 0.25 mg/L [52]. *C. dubia* was also exposed to MWCNT of three diameters (14, 35 and 60 nm) dispersed by three treatments (ozone and ultrasound, ultrasound only, or mechanically dispersed) in an 24 h acute mortality assay [81]. There was no difference in CNT toxicity based on primary particle size; rather toxicity was governed by size of aggregates influenced by surface treatment. Sonication treatment of MWCNT induced highest *C. dubia* 24 h mortality with LC50 between 2–8 mg/L, compared to LC50 8–20 mg/L after stirring, and LC50 100 mg/L after ozone/ultrasound treatment [81]. In the same study, 60 nm ozone or sonication treated MWCNT were tested in the 3-brood reproduction assay. Sonication treated MWCNT affected the population growth more than ozone treated MWCNT at EC50 4 mg/L and 17 mg/L, respectively [81]. Ozone treatment clearly oxygenated MWCNT surface and reduced the toxicity. In two studies, Kennedy et al. investigated the toxicity of functionalized MWCNT influenced by different dispersion protocols in an 48 h acute mortality bioassay with *C. dubia*[55,57]. The raw MWCNT dispersed in NOM were more toxic to *C. dubia* than functionalized MWCNT with hydrophilic groups (hydroxylated or carboxylated), mortality at LOEC 16 mg/L and 48 mg/L for the raw or both functionalized MWCNT respectively. Other functionalized MWCNT (alkylated, aminated) were more toxic to *C. dubia* compared to the raw MWCNT, causing increased mortality at LOEC 15 mg/L and 2 mg/L. Dispersion by stirring or sonication did not induce major changes in toxicity, after sonication a minor decrease in toxicity was observed in *C. dubia*. The toxicity was reported despite rapid settling process (sediment is the repository), though functionalized groups and the presence of NOM slowed down the settling process [55,57]. Reproductive toxicity was assessed by exposing *C. dubia* to MWCNT dispersed by sonication in reconstituted water with NOM [132]. After 7d (reaching three broods) was observed 20-22% reduction in reproductive means at LOEC 2.38 mg/L (pH 6;8) or 4.77 mg/L (pH 7) [132]. Compared to *D.magna*, *C.dubia* is less sensitive to MWCNT exposure, possibly due to a higher reproductive rate. Thus, similarly to *D. magna*, MWCNT induced mortality in *C. dubia* based on surface treatment and dispersion protocol. The LC50 ranged from 2–100 mg/L, while developmental and reproductive effects were induced from 0.2-17 mg/L. Sonicated CNT induced generally effects at lower concentrations, compared to stirred CNT.

Similarly to results observed with *C. dubia*, the choice of dispersion method influenced toxicity in a harpacticoid copepod *T. japonicus* exposed to DWCNT dispersed either by stirring or sonication in a life cycle test [82]. The stirred DWCNT were less toxic compared to the sonicated ones. Larval mortality was observed at LOEC 100 or 30 mg/L and population growth inhibition at LOEC 0.1 or 10 mg/L, for stirred or sonicated DWCNT, respectively [82]. As observed in all *Daphnia* studies, *T. japonicus* mortality was induced at higher concentrations compared to subchronic exposures during population growth evaluation.

Few other reports are available on the toxicity of CNT in soil dwelling invertebrates. A free-burrowing estuarine copepod *A. tenuiremis*, was exposed to SWCNT in a bioassay assessing acute and chronic life-cycle effects [83]. SWCNT dispersed in sea water were assessed as raw, or electrophoretically purified, or as a fluorescent fraction of nanocarbon synthetic by-products. The raw SWCNT induced mortality, reduced fertilization and molting success with a LOEC of 10 mg/L, while the fluorescent fraction of nanocarbon synthetic byproducts induced mortality with a LOEC of 10 mg/L and reduced molting success with the LOEC of 0.58 mg/L. In contrast to raw SWCNT and fluorescent fraction of nanocarbon synthetic byproducts, purification eliminated SWCNT toxicity with NOEC 10 mg/L for all parameters [83]. Another sediment living infaunal marine organism, lugworm *A. marina*, was exposed to SWCNT sonicated and dispersed in a sea water/sediment mixture for 10d [84]. No significant effects on burrowing behavior or cellular and DNA damage in coelomocytes (free somatic cells) were observed (NOEC 0.03 g/kg) [84]. A study with sediment dwelling organisms freshwater amphipod *Hyalella Azteca,* midge *Cironomus dilutus,* oligochaete *L. variegatus* and mussel *Villosa iris* assessed toxicity of SWCNT and MWCNT in 14d water-only 1 g/L exposures [133]. The focus of the study was the effect of CNT pretreatment (sonication and acid washing) on toxicity. While acid washing removed metal content from the CNT surface and reduced the toxicity compared to pristine CNT, sonication effect was less clear and dependent on species [133]. Two studies assessed MWCNT dispersed by stirring, sonication or sonication in NOM in acute whole-sediment assays with marine amphipod *Leptocheirus plumulosus* and freshwater amphipod *Hyalella azteca*[55,57]. MWCNT treated by all dispersion protocols induced mortality of *L. plumulosus* at 30 g/kg, while only sonicated MWCNT induced mortality of *H. azteca* at 300 g/kg [55,57]. Thus, different dispersion protocols may modulate sediment toxicity.

It could be anticipated that sediment will be an important sink of CNT and therefore the exposure may have important environmental implications. It is however difficult to predict whether the observed effects in sediment dwellers are environmentally relevant, since effect concentrations change when different dispersion protocols are introduced.

#### Effects on pelagic vertebrates

Target organs for nanomaterial toxicity in fish may include gills, gut, liver and brain [134,135]. The choice of target organs was based on an analysis of founding assumptions in fish physiology and toxicology: 1) nanomaterials may be trapped by the mucus layer in gills, but are unlikely to penetrate the tight junctions between the cells and enter the blood; 2) Fish gut epithelium may be able to take up nanomaterials via endocytosis, especially if particles are lipophilic; 3) Fish skin is especially robust and protected by mucous secretion, thus skin would act as barrier for nanomaterials; 4) nanomaterials may also enter fish system via buccal cavity, olfactory openings, eyes, and urinary openings. These cavities are however well protected; 5) Large nerves (e.g. olfactory nerve) are in a close proximity to fish cavities, thus nervous system may be exposed. Mechanistic damage to nerves or brain and neurotoxicity may lead to alterations in fish behaviour, e.g. aggressive behaviour observed [136]. Generally for CNT, fish translocation studies are still lacking, however nanoparticle-induced inflammation can cause gill and gut injury [136] and may lead to direct uptake into blood. Liver is reported to be a target organ after gastric exposure to nanomaterials and oxidative stress and liver injury were observed after exposure to SWCNT [136].

Studies with aquatic vertebrates assessed CNT acute and chronic toxicity. Protocols included early life toxicity that is considered to be the most sensitive exposure, as well as exposure after fulfilled development. Early life toxicity was assessed in zebrafish (*Danio rerio*) embryos (4 to 96 h post fertilization) exposed to SWCNT dispersed in tap water [89]. Delayed hatching was observed at LOEC 120 mg/L, while embryonic development was unaffected (NOEC 360 mg/L). In the same study, DWCNT dispersed in tap water delayed hatching at the LOEC of 240 mg/L [89]. In a study with similar design, zebrafish embryos were exposed from 8-72 h post fertilization (OECD 210) to MWCNT dispersed in zebrafish medium [40]. Phenotypic effects were observed at LOEC 60 mg/L, while a dose of 100 mg/L induced significantly delayed hatching and mortality. In the same study, microinjection of 5 ng MWCNT to 8-cell-stage zebrafish embryos (OECD 212) induced comparable effects to the aquatic MWCNT exposures [40]. Microinjection of 2 ng MWCNT to 1-cell-stage zebrafish embryos (acid treated) did not induce mortality or abnormal development in zebrafish up to the second generation (Full life-cycle assay), though a reduced survival was observed in the second generation [90]. Immune response was observed in the early stages of the first generation, suggesting negative effect on the exposed zebrafish [90]. In a follow up study with the same protocol, the MWCNT were cut by sonication in acid for 48 h compared to 24 h (length 200 nm and 800 nm, respectively). The shorter CNT induced severe developmental toxicity, in contrast to the previous study, while the longer CNT did not affect the embryos [137]. Another study supports that the dispersion protocol can influence the CNT toxicity. Fry of medaka fish *Oryzias melastigma* were exposed from 2 d post hatching for 14d to DWCNT suspended in artificial saltwater [82]. Carbon nanotubes were dispersed by sonication or stirring. The tested DWCNT formed smaller aggregates in the sea water after sonication, compared to stirring. After 14 d exposure medaka growth was reduced with LOEC 10 mg/L sonicated DWCNT, while the stirred DWCNT reduced medaka growth at concentration 10-fold higher (LOEC 100 mg/L) [82]. Thus the sonication protocol affects the CNT characteristics such as length and agglomerate size and this way alters the CNT toxicity.

CNT toxicity and the effect of dispersion protocol were also assessed in early stages of African cleaved frog *Xenopus leavis.* Larvae were exposed from developmental stage 50 for 12 d to DWCNT and MWCNT and acute toxicity and genotoxicity were evaluated [64,91-93]. DWCNT were evaluated in series of experiments (with or without aeration and with or without dispersant GA) [91]. Aerated DWCNT were better dispersed compared to DWCNT without aeration, however they were less toxic compared to non-aerated DWCNT, since only minor mortality (5-15%) was observed at 10 mg/L (LOEC) and reduced growth at 100 mg/L (LOEC). In comparison, non-aerated DWCNT induced massive mortality (85%) at 500 mg/L (LOEC) and reduced growth from 10 mg/L. The authors suggested that since DWCNT blocked the gills, the toxicity may have been induced by anoxia in media with less available oxygen [91]. Comparison of the toxicity of DWCNT with and without dispersant GA indicated that adding GA reduces the toxicity. Minor mortality was observed at 10 mg/L (LOEC) without GA, whereas no mortality was observed up to 50 mg/L with GA present. Both experiments observed growth retardation with a LOEC of 10 mg/L [93]. MWCNT dispersed in water without GA (same experimental conditions as with DWCNT) were evaluated for toxicity and genotoxicity [92]. In contrast to DWCNT, MWCNT were less toxic and reduced amphibian growth first at a LOEC of 50 mg/L and did not induce mortality. The authors suggested that the toxicity is likely affected by diameter, since it is more difficult for thicker CNT to enter the organisms [92]. The same research group assessed the effects of dispersion methods with two types of dispersant on the MWCNT toxicity [64]. MWCNT were tip sonicated or mechanically rotated with two dispersants (carboxymethylcellulose MWCNT-CMC or GAMWCNT-GA). The MWCNT were stable up the 24 h media change in the presence of dispersants, while raw MWCNT sedimented during this time. Therefore the MWCNT toxicity in the presence of dispersant was more severe (LOEC mortality 50, 1 or 50 mg/L and LOEC growth inhibition 50, 10 and 10 mg/L, respectively). MWCNT-CMC were more toxic than MWCNT-GA. The authors suggested the GA is a potential antioxidant that modulates the effects. MWCNT-CMC induced genotoxicity evidenced as micronucleated erythrocytes at LOEC 1 mg/L [64], while other CNT exposures did not [64,91-93]. Similarly as discussed in studies with zebrafish, the CNT treatment and dispersion protocol affects the final CNT toxicity. Better dispersion may induce higher toxicity, however surface treatment may also reduce the possible effects.

Two studies assessed effects of SWCNT in an older fish, juvenile rainbow trout *Oncorhynchus mykiss*[136,138]. An exposure under semistatic conditions 10d to SWCNT induced dose-dependent systemic toxicity in *Oncorhynchus mykiss* starting at 0.1 mg/L, in absence of oxidative stress or hematological changes. Exposure caused respiratory toxicity, neurotoxicity and hepatotoxicity [136]. In contrast, a dietary exposure of 500 mg/kg SWCNT twice a day for six weeks, followed by two weeks recovery, did not result in obvious toxicity (weight, hematological alternations, metal accumulation, oxidative injury or pathology). A transient elevation of thiobarbituric acid reactive substances indicative of lipid peroxidation present in the brain was observed after the SWCNT exposure [138]. Thus, the exposure route may considerably affect the overall CNT toxicity. Also, when other parameters than mortality and growth are assessed, the sensitivity of this exposure time may exceed the early stage exposures.

In conclusion, vertebrate species respond to CNT exposure at relatively higher concentrations compared to the invertebrates, even though the exposure protocols tend to use the most sensitive stage of the developing embryos and larvae. The most sensitive end point in the vertebrate studies was assessment of respiratory toxicity in juvenile trout, were effects were observed at doses as low as 0.1 mg/L. All studies suggest that CNT type, treatment and dispersion protocol will affect the CNT toxicity.

### Effects on terrestrial species

#### Effects on terrestrial invertebrates

Only few terrestrial invertebrate species were investigated for effects of CNT. DWCNT were added to dry food in a 28d sub-lethal toxicity assay with soil dwelling earthworm *Eisenia veneta*[139]. Concentrations up to 495 mg/kg dry food did not induce mortality or reduce *E. veneta* weight, and second generation hatched and survived normally. However, already at 37 mg/kg dry food the number of produced cocoons was reduced by 10%, suggesting that DWCNT may affect earthworm reproduction [139]. In a series of bioaccumulation studies with SWCNT and MWCNT, no mortality of *Eisenia foetida* was observed in concentrations up to 3000 mg/kg soil [69,87,88]. Thus *E. foetida* may not experience acute toxicity due to contact with CNT containing soil, although effects of chronic exposures still have to be determined.

The fruit fly *Drosophila melanogaster* was assessed in three studies for acute and reproductive toxicity from the larval stage to adult stage [94,95,140]. *Drosophila* fed SWCNT spiked dry yeast at 9 mg/L survived and developed normally, despite the CNT transfer in body compartments [94]. Similarly, *Drosophila* fed SWCNT and MWCNT spiked larval gel food at concentrations up to 1 g/kg food hatched and survived normally [95]. However, when *Drosophila* was exposed directly to the nanoparticle powder, CNT adhered to the body, reduced grooming behavior, impaired locomotor function and induced mortality [95]. When the fruit fly *Drosophila melanogaster* was exposed to hydroxilated SWCNT dispersed by sonication in water at concentrations 0.005-0.5% w/v, no effect on fecundity or fertility was observed [140]. Thus, CNT did not induce acute or reproductive toxicity to *Drosophila*. The only toxicity was induced by direct contact with CNT, which is unlikely in the natural settings.

#### Effects on terrestrial vertebrates

No studies are available with wild terrestrial species. A large number of laboratory rodent studies with inhalation route and injection exposure route are available, as reviewed by [17,18]. For the purpose of this environmental review, only the laboratory studies with oral exposure will be mentioned, as the most probable environmental exposure. When female Fisher rats were given an oral gavage to 0.064 or 0.64 mg/kg SWCNT in saline or in corn oil, increased levels of oxidative damage to DNA in liver and lung tissue were observed [141]. Thus CNT ingestion may be genotoxic to terrestrial mammals. Reproductive toxicity of SWCNT was assessed after ingestion of 10 mg/kg dispersed by sonication in tragacanth gum solution to a CD-1 mouse on gestation day 9 [140]. Exposure induced resorption, gross morphological defects, and skeletal abnormalities, without effect on litter size or maternal or offspring weight. Ten times higher dose (100 mg/kg) did not induce any adverse effects, and authors propose that this was due to greater agglomeration in the gavage suspension [140]. In a Sprague–Dawley rat study, oral administration from gestation day 6 to 19 to dose up to 1000 mg/kg/day (14000 mg/kg total dose) did not induce teratogenicity [142], despite an effect on the immune function evidenced by reduced weight of maternal thymus. Whether CNT can induce genotoxic and reproductive effects in mammals have to be further investigated and this topic is beyond the scope of this paper.

#### Effects on terrestrial plants

Few phytotoxicity studies report no effects or effects at relatively high doses of CNT, using modified U.S. EPA Test 7101 or OECD Guideline 206 (reviewed in [143]). In a germination study, six plant species seeds (radish, rape, ryegrass, lettuce, corn and cucumber) were soaked and germinated for 5d in 2000 mg/L MWCNT sonicated in deionized water [144]. No difference in seed germination or root growth was observed [144]. Zucchini *Cucurbita pepo was* exposed for 15 d to 1000 mg/L MWCNT sonicated in 25% Hoagland media, had normal germination and root elongation but reduced the plant biomass by 60% compared to controls [145]. Also seven crop species (lettuce, rice, cucumber, spinach, lady’s finger, chili, and soja) were exposed as seedlings for 15d to 20–2000 mg/L MWCNT in Hoagland media [146]. Phytotoxicity was observed for spinach, lettuce, rice and cucumber (in sensitivity order) at LOEC 1000 mg/L, while lady’s finger, chili and soja did not respond to the exposure at NOEC 2000 mg/L [146]. In a follow up study, new types of MWCNT were tested with the most sensitive species identified (spinach). The observed phytotoxicity was ROS induced (LOEC 125 mg/L) [147].

Some studies also present plant growth stimulation by contact with CNT. Six crop species (cabbage, carrot, cucumber, lettuce, onion, and tomato) were exposed as seedlings for 24 or 48 h to 56–1750 mg/L non-functionalized and functionalized SWCNT [98]. Generally, the root elongation was more affected by non-functionalized SWCNT (with both enhancing and inhibitory effects, at low and high dose respectively). Effects early after exposure (24 h) were more pronounced than the longer (48 h) exposure [98]. Pristine or oxidized MWCNT sonicated in deionized water enhanced (23 or 2.3 mg/L, respectively) or inhibited (46 or 6.9 mg/L, respectively) mustard germination and growth up to 10d [101]. It was suggested that the positive effect was due to increased water intake, while the higher dose was phytotoxic [101]. Similarly, MWCNT dispersed in a growth medium at concentrations of 10, 20 or 40 mg/L enhanced seed germination and biomass production [100]. The authors suggested that CNT promote water transport inside the seeds by penetration of the seed coat or by the regulation of water channels [100]. This hypothesis was confirmed in the follow-up study, where tomato seeds were germinated and grown in medium with 50 mg/L carboxylated SWCNT or MWCNT dispersed in a growth medium [102]. The CNT enhanced plant growth and a microarray analysis indicated that the gene expression was altered for stress related genes (similar to pathogen response) and water channel genes [102]. When a specific water channel protein (LeAqp1) was analyzed in roots of 8 or 41d old tomato seedlings germinated and grown in the presence of 40 mg/L MWCNT, higher LeAqp1production was detected in plants that exhibited increased germination and growth [102]. Growth enhancement was observed for four MWCNT modified by acid dispersion, while the agglomerated pristine form had no effect [104]. Thus, the growth enhancement is dependent on CNT plant interaction.

To summarize, soil is expected to be a sink of CNT and therefore terrestrial toxicity assessment is important. The presented studies generally do not find effects even at high concentrations. The soil dwelling earthworm *E. veneta* does not respond by mortality or growth reduction to high doses of CNT [69,87,88], however minor effects on reproduction (cocoon production) were observed [139], suggesting that effects of low chronic doses need still to be evaluated. In rodent studies, oral exposure to CNT induced genotoxicity [141] and results for reproductive toxicity are contradictory [140]. When plant toxicity is evaluated, studies generate complex results. Some studies suggest that CNT in low doses can penetrate into roots and seeds and stimulate plant growth [98], while other studies do not observe this effect or report phytotoxicity at high doses [101] . Many studies use hydroponic models to simplify testing. However, more results in soil are desirable, because they take into account the soil complexity. More standardized studies assessing plant toxicity are needed, to establish the mechanisms and conditions of CNT phytotoxicity.

## Hazard identification

Environmental risk assessment of nanomaterials requires thorough characterization of nanoparticles subjected to risk evaluations and suitable methods are needed for determining the realistic environmental concentrations in complex matrices [7]. The first ecotoxicological studies were published almost ten years ago, but to date there is still a limited number of high quality data available for hazard assessment of nanomaterials [10,16,148]. It has currently been impossible to group nanomaterials on the basis of inherent properties, since mechanisms of toxicity are not yet well defined or understood. Some consensus has been reached for CNT, where shape, size and aspect ratio are key parameters for hazard identification in relation to humans [149] and may also serve as a minimum requirement for environmental hazard identifications. Though, cause-effect relations for CNT are not well documented for these properties in environmental organisms. As defined by REACH, the persistence, bioaccumulation, toxicity profile (PBT-profile) is of major importance for environmental hazard identification of nanomaterials. Thus, a strong focus should be directed to ecotoxicity, biodegradability, mobility (uptake), bioavailability, and ultimately processes possibly leading to bioconcentration and/or biomagnification [149].

We have summarized literature available on biological fate, and effects of CNT in organisms relevant for environmental hazard identification and risk assessment. From the sparse literature, pollution of manufactured CNT in the environment has not been reported [41-43]. The CNT may be completely combusted in well-functioning waste incineration plants [44,45], may be prone to limited leaching when products containing CNT are disposed of in landfills, and due to the use pattern of CNT in products (incorporated in a matrix), little is expected to be discharged via municipal sewage treatment effluent [32,47-49]. However, CNT are of an industrial interest in a wide range of areas and therefore the production [22], use and environmental release will increase dramatically. It is of concern, since CNT are biopersistent pollutants and the effects are still largely unknown. Moreover, CNT have a great pollutant adsorption capacity and may in the future be explored for bioremediation purposes [33-38,53,65-72]. This potential application calls for careful risk-benefit analysis prior to large-scale implementation [150]. For example, sediment loading higher than 1.5 wt% CNT had low sorption efficiency towards PAH and increased accumulation in the benthic *Chironomos plumosus* larvae with toxic effects [73,74]. In addition, one should also consider the occupational and environmental exposure to humans, in which the potential hazards of CNT are of very great concern, with currently proposed exposure limits down to 1 μg CNT-related carbon per m^3^[151]. Consequently, the added value of using CNT for remediation as a substitute for other known compounds may be limited.

Environmental behavior of all types of CNT greatly depends on the surface properties and environmental conditions they are released into. Pristine CNT are more reactive but they tend to be difficult to disperse in water due to their hydrophobicity, poor solubilization and often entangled or aggregated nature. Their stability in dispersions, like any other material, also strongly depends on water pH and ionic strength. In experiments with dispersant sodium dodecylbenzene sulfonate stabilized CNT dispersions, agglomeration was greater at decreased pH and increased ionic strength [152]. Poorly dispersed powders and formation of agglomerates can increase the settling rate of CNT. Therefore, the sediment is expected to be the greatest environmental sink for CNT. However, modified surface properties created intentionally or via natural coatings by e.g. natural organic matter may still and prolong residence time of CNT in the water column, depending on the pH and ionic strength. Surface properties would play important role in CNT adsorption capacity and selective adsorption may be achieved by specific surface modifications. However, CNT released accidentally into the environment may also adsorb other pollutants and give rise to combination effects [153]. Thus, exposure to pelagic and bentic species can be expected and the aquatic food web would be affected [1] and CNT could enter the human food chain via fish [1].

As discussed in section 5, CNT do not cross readily from the body surface into invertebrate animals. During vertebrate development, CNT have not been found to enter the embryo and if internalized into the blood stream, CNT are cleared rapidly [89,90]. When ingested, CNT seem generally to be fully excreted [91-93]; or only a small fraction may translocate into blood and internal organs [94,95]. Whether CNT are taken up from the digestive system can depend on CNT type (SWCNT or MWCNT) and size, as observed in rodent studies [96,97]. An almost full excretion was observed for mice fed MWCNT, whereas a high degree of translocation was observed in mice fed very small SWCNT [97]. However, it should be noted that CNT detection in biological tissues is a challenge. It is possible that CNT translocation determined by electron microscopy is underestimated, or detection via a radioactive labeling on the CNT surface may be overestimated. It can be speculated, that MWCNT present in the gut of lower organisms would also be excreted via defecation, without uptake when ingested by higher species, whereas ingestion of lower organisms containing small SWCNT could lead to a CNT biomagnification in the food chain. It has been shown that CNT presence on or in the organism may induce toxicity, by reducing organism food intake by gut clumping [52,77-79], impeding mobility [57] or inducing other physiological reactions to CNT presence (e.g. oxidative stress, lipid peroxidation) [91-93,95,126,136,147]. Binding between the CNT and body tissues is modified by functional groups on the CNT surface. In plants, CNT can penetrate seeds during germination and roots during growth. This penetration can affect the plant physiology and alter the uptake of water and nutrients. This could affect the plant growth positively [100-102,104] and negatively [101]. In some studies CNT are transferred from roots further into leaves and fruits [100-102], while in others CNT stayed on the outer seed or root surface or the transfer was minimal [75,98,103,144]. Differences possibly depend on the type of exposure.

Based on the current data, it is difficult to reach consensus on the toxicity of CNT in aquatic and terrestrial organisms. The current studies analyze many different types of CNT, with different lengths, different surface treatments, and dispersed by a variety of protocols. Often only the nominal concentrations are presented and the behavior and fate (e.g. sedimentation) of the tested CNT is unknown. In the future studies, inclusion of benchmark materials with well-known biological effects could aid in proper inter and intra interpretation of the assessed toxicity. While better material characterization *per se* is essential for increasing the understanding of the exposure/effect relationship, the lack of knowledge about CNT mode-of-action hampers firm conclusions on cause-effect relationships between inherent properties and toxicity.

In bacteria, SWCNT are generally more toxic compared to MWCNT [110,114,117,118]. The length of the CNT has been found to affect the toxicity [111]. Some authors report that shorter CNT are more toxic due to increased interaction with bacteria [105], thus damaging the bacterial membrane to a higher extent [113]. Surface modifications affect the toxicity by modifying the surface reactivity and the ability to aggregate with bacteria [108,110]. Suspension media can both facilitate and inhibit the CNT toxicity [105,110]. Increase of toxicity could be due to a more complete dispersion of CNT, while reduced toxicity could be caused by a protein coating of the CNT surface thereby reducing reactivity.

**Figure 2:**
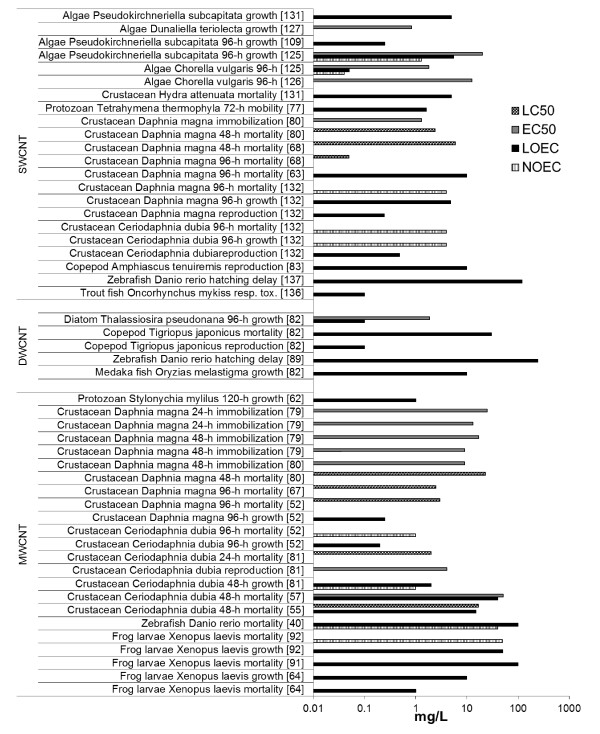
**Dose descriptors for aquatic toxic effects in pelagic species.** Worst case scenario from all articles. LC50: lethal concentration 50%; EC50: effect concentration 50%; LOEC: lowest observed effect level; NOEC: no observed effect level.

For aquatic organisms, a wide range of CNT dispersion protocols have been tested, in order to identify the best protocol available for aquatic toxicity studies [55,57,81,82,109,125-127,132]. Generally, protocols leading to a more complete dispersion of CNT result in a higher level of CNT/organism interactions and thereby increase the toxicity. Agglomerates tend to settle fast. Therefore poorly suspended CNT often settle before affecting the tested pelagic aquatic organism. This could be the reason why sonicated CNT induce toxicity to aquatic organisms at lower concentrations compared to stirred CNT [55,57,81,82,84,132,133]. It was however suggested, that sonication can increase the availability of metallic impurities, thus possibly increase the toxicity in the solution [154]. Moreover, less stable CNT may be cut when using high energy sonication input for dispersal. The use of various suspension media may similarly facilitate or inhibit CNT toxicity . Depending on the proportion of dispersant in the suspension media, dispersant would aid to better CNT dispersion, but it could also coat the CNT and reduce the observed effect [35,38,52,55,57,64,66,67],[91-93,109,110,114,120,132]. Based on the data presented in Figure 2, lower pelagic organisms such as algae and daphnids are more sensitive to CNT compared to vertebrate organisms such as fish and frog larvae. Most pronounced effects are generally observed after prolonged exposures (e.g. 96 h exposure for algae and daphnids); and for endpoints assessing functions (growth, mobility, reproduction) rather than mortality. In the reviewed studies, SWCNT induced effects in lower organisms at concentrations from 0.05-10 mg/L [63,68,77,80,83,109,125-127,131],[132], while DWCNT and MWCNT induced effects at concentrations from 0.1-51 mg/L [52,55,57,78-80,82]. Toxicity of CNT to aquatic vertebrates ranges from 10–240 mg/L [40,64,82,89,91-93,136], although SWCNT were found to be a respiratory toxicant to trout fish starting at 0.1 mg/L [136]. This would classify the CNT toxicity according to the European Union Commission Guideline 93/67/EEC, introduced for nanoparticle toxicity by [131], as extremely toxic to harmful for the lower aquatic species, and very toxic or harmful to not toxic for aquatic vertebrates presented in Table 3.

**Table 3:** CNT aquatic toxicity

		**Extremely toxic**	**Very toxic**	**Toxic**	**Harmful**	**Not toxic**
		**(<0.1 mg/L)**	**(0.1-1 mg/L)**	**(1–10 mg/L)**	**(10–100 mg/L)**	**(>100 mg/L)**
**SWCNT**	**invertebrates**	X	X	X		
	**vertebrates**		X			X
**DWCNT**	**invertebrates**		X	X	X	
	**vertebrates**			X		X
**MWCNT**	**invertebrates**		X	X	X	
	**vertebrates**				X	X

The CNT aquatic toxicity classified according to the European Union Commission Guideline 93/67/EEC, introduced for nanoparticle toxicity by [131].

**Figure 3:**
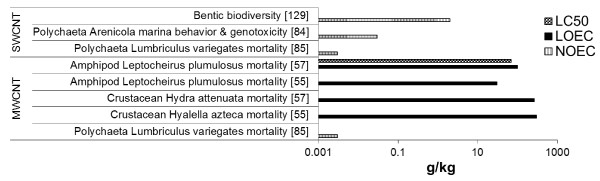
**Dose descriptors for aquatic toxic effects in benthic species.** Worst case scenario from all articles. LC50: lethal concentration 50%; LOEC: lowest observed effect level; NOEC: no observed effect level.

The few studies performed on CNT toxicity to benthic organisms are summarized in Figure 3. Benthic toxicity was only observed at high concentrations [55,57], the lowest adverse effects observed at concentrations 30 gCNT/kg sediment [57].

**Figure 4:**
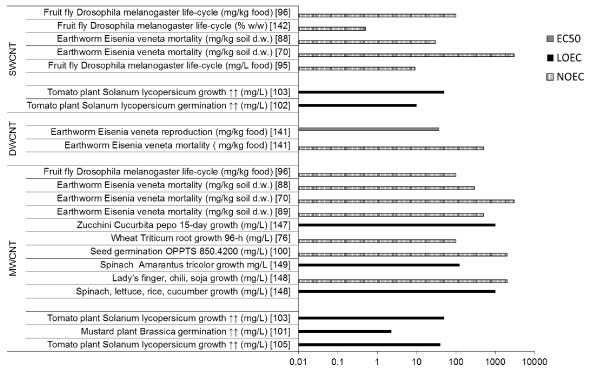
**Dose descriptors for terrestrial toxic effects.** Worst case scenario from all articles. EC50: effect concentration 50%; LOEC: lowest observed effect level; NOEC: no observed effect level.

Terrestrial toxicity is an important aspect of CNT risk assessment. Similarly to effects observed in the sediment, adverse effects in the soil were induced at high concentrations in mg CNT/L exposure media [145,147], presented in Figure 4. Studies were performed as hydroponic cultures and therefore the observed CNT effect are presented in mg/L concentration. A minor reproductive effect on earthworm was observed after exposure via food at concentration 37 mg/kg [139]. Based on the current reports, the effects on terrestrial organisms are unlikely.

The physical and chemical characterization methods used in the reviewed toxicological articles are presented in Table 2. The characterization data analysis supports that the CNT types included in this review were different. The CNT differ in the number of walls and different properties. In addition, some of the samples contain catalyst materials. The included toxicological tests were performed on both pristine and purified samples. The purifications differ depending on the intended use of the CNT, e.g. purification for removing catalyst, for improving the quality, or for removing a certain subtype of tubes. The pristine CNT are often inhomogeneous when subsampled and therefore full characterization can be expensive and time consuming. A well purified sample tends to be more homogenous and therefore it needs less characterization before obtaining representative information about the sample.

It has become a common knowledge that CNT characterization can differ from manufacturer data and between subsamples. Therefore it has become a standard practice to characterize the samples before use. Only few articles in this review were not stating any characterization or stated only the information from the manufacturer. Most articles included some CNT characterization. Especially characterization of diameter, length, description of surface area and agglomeration were commonly included.

The CNT can be characterized as a powder, or in the stock solution, or in the final concentration in the exposure media. In the reviewed articles, the CNT were usually characterized as a powder or in the stock solution. It was not always possible to distinguish in what form the CNT were characterized. Characterization of the CNT in the exposure medium tends to be more complicated, because CNT characteristics change over time, e.g. agglomeration occurs. The possible changes during the exposure at actual concentrations should be however addressed, to fully explain the observed biological effects. For the purpose of aquatic toxicology it is important to note, that not all characterization techniques are suited for characterization of CNT in liquid. Though the full and true knowledge of the CNT may not be obtainable, important information can still be gained. The dynamic aspect of exposure from dosing to target is however essential, to assess the fate in the ecotoxicological assay. The preparation techniques are known to have an effect on CNT, as discussed for sonication and dispersants. It is the authors general opinion that the characterization of the true exposure is equally important as the characterization of the raw material.

## Conclusion

CNT are a large group of carbon-based, tube-like nanomaterials, which not only differ in length and the number of layers they consist of, but also vary in types of impurity, their contents and surface modification. In the reviewed studies, a variety of CNT from different sources with different compositions were used. The CNT were suspended in variety of media and with an assortment of dispersion protocols. However, some general conclusions about CNT toxicity can be drawn from the reviewed studies.

The changes in surface properties or the adsorption to other compounds determined significantly CNT environmental behavior. Generally it is assumed that hydrophobic pristine CNT are poorly dispersed and will agglomerate in water and sediment to the benthic zone. Pristine CNT would sediment faster than functionalized hydrophilic CNT. These differences may influence both the behavior in the environment, in aquatic ecotoxicological tests, as well as the interaction with organisms in general.

Like other carbon based materials, e.g. activated carbon, CNT have strong sorbent properties, which can be used intentionally in e.g. remediation applications but may also bind compounds present either in the environment or in test media. Both, natural organic matter and various pollutants bind to CNT. When a mixture of organic compounds and pollutants were applied to CNT solution, the binding followed in a competitive manner [37]. The presence of CNT in the environment may also increase the bioavailability of free metal ions due to the sorption of NOM in competition with the metal ions [66]. These sorption processes change the surface properties of CNT, their behavior in the environmental media and finally CNT toxicity.

CNT behavior in ecotoxicological test media will be influenced by their property characteristics, the media type, and the dispersion method. How this may influence the interaction of CNT with organisms is hardly predictable. Hence, in future studies it is necessary to include an extensive exposure characterization, consisting of a chemical characterization followed by a careful assessment of interactions with the test media. Two factors need more attention in the future, the sorption processes and the effects of dispersants. The CNT sorption may alter the composition of the ecotoxicological test media by binding components of the media. This may lead e.g. to a reduction of nutrients in the medium, or in case the CNT are ingested, to an increased nutrient uptake by organisms. This may explain the observed stimulatory effects at low concentrations. The use of dispersants and the application of various dispersion methods has to be clearly defined and characterized in each test, with a differentiation between the initial dispersal (e.g. in water) and the subsequent dispersal in test media (e.g. *Daphnia* media). CNT will behave differently in different media and the CNT toxicity may be influenced when using high energy sonication input for dispersal.

The behavior of CNT in the different media influences also the uptake and bioaccumulation by organisms. In general, an uptake of CNT into organisms was observed, which was normally followed by a rapid elimination in both aquatic (daphnids) and soil organisms (earthworms, plants), and in invertebrates and vertebrates alike. However, no or only marginal transfer of CNT into tissues was observed. As an uptake of CNT was observed especially in primary consumers like daphnids, CNT biomagnification is an issue of high relevance for the future. Since the detection of carbon based materials in organisms remains a challenge, it is unclear how valid are the results generated by various approaches.

However, CNT present in or on the body may induce toxicity, which is related to surface area with SWCNT being more toxic to organisms than MWCNT. Also, CNT length and dispersion degree play a role for the toxic outcome. It can be assumed that the ratio length/diameter is an important factor; however, this was not systematically assessed so far. Hence, the fiber or tube shape plays an important role in toxic outcome, leading to indirect and direct effects on organisms. Direct mechanical effects were observed in bacteria, fish and plants, were the CNT pierced and consequently damaged cells. Indirect mechanical effects were observed e.g. in *Drosophila* and *Daphnia*, where an interaction with the outer surface of animals occurred, leading to interference with movement, grooming behavior and food intake. In general, for the assessment of ecotoxicological effects of CNT, more targeted approaches are needed. The exposure scenario and exposure route has to be derived from the CNT application, use of stabilizers or surface modifications. Here, two scenarios are possible. When designing future studies, two test scenarios are possible. First, the CNT are stabilized in well-defined test system, where dispersants may be acceptable to gain uniform exposure. Second, more environmentally relevant scenario, agglomeration may be accepted and dose derived from nominal concentrations. The exposure characterization is an essential part of result reporting.

In summary, from the data presented in the reviewed studies CNT were identified to be hazardous to aquatic organisms, with SWCNT being more toxic than DWCNT and MWCNT, and invertebrates being more sensitive than vertebrates. All observed effects were evident at concentrations higher than environmental concentrations presently predicted for water, sediment and soil. These estimates are however highly uncertain and as a minimum more robust data on production volumes are needed, to give better predictions on environmental concentrations.

## Abbreviations

1 h: 1 hour; 1d: 1 day; 1m: 1 month; CNT: Carbon nanotube; SWCNT: Single-walled CNT; DWCNT: Double-walled CNT; MWCNT: Multi-walled CNT; COOH-CNT: Carboxylated; OH-CNT: Hydroxylated; HN2-CNT: Amino functionlized; PEI-CNT: Grafted with polyethylenimine; NOEC: No observed effect level; EC50: Effect concentration 50%; LOEC: Lowest observed effect level; LC50: Lethal concentration 50%; pKa: An acid dissociation logarithmic constant; HOC: Hydrophobic organic contaminants; PAH: Polycyclic aromatic hydrocarbons; BSA: Bovine serum protein; GA: Gum Arabic; DOM: Dissolved organic matter; NOM: Natural organic matter; CMC: Carboxymethylcellulose; ROS: Reactive oxygen species; EPA: Environmental Protection Agency; OECD: Organisation for Economic Co-operation and Development.

## Competing interests

The authors declare that they have no competing interests.

## Authors’ contributions

PJ searched and evaluated all literature, drafted the manuscript. RB reviewed and analyzed all literature for physical and chemical characterization. NRJ, AB, RB, DK, KAJ, UV, HW contributed to defining the scope and structure of the manuscript, contributed to part of the text. AB, DK further supported the literature search and evaluation, and drafted the concluding chapters. All the authors revised critically and approved the final manuscript.

## Supplementary Material

Additional file 1: Table S1 Overview of nanoecotoxicology literature. Click here for file
