# Supplementary material for: Bioaccumulation and ecotoxicity of carbon nanotubes
Source: Chem Cent J. 2013 Sep 13;7:154. doi: 10.1186/1752-153X-7-154 (PMC3848800; doi:10.1186/1752-153X-7-154)
Supplement: Additional file 1: Table S1 — Overview of nanoecotoxicology literature. [file 1752-153X-7-154-S1.doc]

BIODISTRIBUTION

| **Particle** | **Characterization**  **method used** | **Model organism** | **Exposure condition, duration**  **and detection method** | **Conclusions** | **Ref** |
| --- | --- | --- | --- | --- | --- |
| SWCNT  (HiPCo tube, Carbon Nanotechnologies)  Refluxed in HNO3  2-10 nm x >500 nm  Mixed in a complex ciliate growth medium, proteose peptone extract. | AFM  SEM  EDAX  After:  SEM | *Tetrahymena thermophila*  Unicellular ciliated protozoa | Chronic toxicity test (72-h)  Bacterivory bioassays  Concentrations 0-17.2 mg/L | SWCNTs were internalized by the ciliates and eliminated again. SWCNT could move up the food chain after ciliate ingestion by other animals. | [1] |
| MWCNT  (method by Shenzhen Nanotech Port Co., Ltd., China)  95% pure (0.1% Ni, 0.2% Fe)  60-100 nm x 600 nm  Treated with AEDP  Dispersed in pond water medium and sonicated | Raman 632.81 nm  SEM  AFM  TEM  ICP-MS  FTIR  After:  Mikroskop 90X  Fluorescens mikroskop  EM | *Stylonychia mytilus*  Unicellular protozoan | Uptake  Concentrations 0.1-200 mg/L | Ingested MWCNTs. | [2] |
| SWCNT  >60% pure  2 nmx5-15 µm  MWCNT  >98% pure  10-20 nm x 5-15 µm  (Shenzhen nanotech Port Co., China)  Suspended in reconstituted water, shaken at room temperature | Manufacturer data  After:  Microscopy | *Daphnia magna*  zooplankton | 48-hour acute toxicity test  (modified OECD Guideline 202)  Concentrations ranging from 0.1-100 mg/L  (not fed during test) | *D. magna* ingested the CNT. | [3] |

| **Particle** | **Characterization**  **method used** | **Model organism** | **Exposure condition, duration**  **and detection method** | **Conclusions** | **Ref** |
| --- | --- | --- | --- | --- | --- |
| MWCNT  >95% pure  (not commercially available)  25 nm x 50 µm  Suspended in 3 different types NOM suspensions (0.2 µm filtered), sonicated for 25 min, diluted and sonicated for 1h, after 24h settled sediment was removed. | DLS  Zetasizer  SEM  IR  After:  TEM | *Daphnia magna*  Fresh water flea | Gut elimination assay  (24h exposure, 2, 4, 8, 12, 24, 48h elimination)  Fed and not fed | MWCNT elimination ↑with food.  Time-to-elimination 2-4 h fed vs. 12-28 h not fed.  Presence of NOM had no effect on time-to-elimination.  MWCNTs were clotting the gut, interfering with food processing and attributing to toxicity.  MWCNTs were unable to adsorb across the gut lumen. | [4] |
| HiPCO SWCNTs  (Carbon Nanotechnologies Inc., TX)  85% pure  1.2 nm diameter  Solubilized by Lysophophatidylchlorine LPC | Spectrophotometry  TEM  After:  Micro-Raman 514.5 nm  Fluorescent microscopy | *Daphnia magna*  Fresh water flea | Uptake  0.1, 0.25, 0.5, 1, 2.5 mg/L | At low concentrations up to 0.5 mg/L Daphnia ingested LPC-SWCNTs, digested LPC coating (used as food source).  Ingested and eliminated SWCNTs were no longer water soluble and they precipitated and sedimented.  Thus, Lipid solubilised NTs may not be stable at biologically rich environments. Organisms would facilitate the removal of LPC,and subsequent clumping into insoluble agglomerates. | [5] |
| MWCNT | TGA  Raman  TEM  Surface area by Micromeritics ASAP 2020  HPLC  Radioactive labeling +  liquid scintillation counting | *Daphnia magna*  Fresh water flea | 24h ingestion and defecation study  Concentrations 0.4 mg/L | MWCNT do not absorb to *D. magna* tissues.  Elimination was possible only in presence of food. | [6] |

| **Particle** | **Characterization**  **method used** | **Model organism** | **Exposure condition, duration**  **and detection method** | **Conclusions** | **Ref** |
| --- | --- | --- | --- | --- | --- |
| SWCNT and MWCNT  (not commercially available)  14C labelled  1) HNO3/H2SO4 (3:1) treated  2) Grafted with PEI (polyethylenemine)  Dispersed in artificial fresh water, sonicated 1h, settled 24h. | NMR (1H)  Zeta potential  TEM  Biological oxidation and measured radioactivity | *Daphnia magna*  Fresh water flea | Uptake and elimination experiments  0.025, 0.25 mg/L | PEI coating improved stability in aqueous solution, but aggregated in d. Magna gut similarly to 3:1 CNTs. Accumulation and elimination was also similar for both CNTs.  Elimination was possible only in presence of food (similar to other studies!!!!). | [7] |
| MWCNT  1) 14.1 nm  2) 34.6 nm  3) 59.2 nm  Dispersed in water  a) O-MWCNTs Treated by ozone 0.14g/min in ultrasound bath for 24h  b) UN-MWCNTsStirred for 1h and sonicated for 60 min  c) Untreated-MWCNTs Stirred for 24h | TEM (ref)  XRD (ref)  TOC (ref)  DLS  Microscope  Zeta potential | *Ceriodaphnia dubia*  Fresh water flea | 24h ingestion and defecation study  Concentration 10 mg/L  Exposures 15, 30, 60 min and 24 h.  Defecation and self-cleaning after 24.  The animals were allowed to cleanse and to rid of MWCNT trapped in  the digestive tracts. | C. dubia ingested and accumulated the nanoparticles in the digestive tract however particles were defecated, indicating the capability of self-cleaning.  US-MWCNT was retained in the digestive tract for a longer time and also required a longer time to be cleaned than O-MWCNT.  Results confirmed the importance of physical–chemical treatment on the interactions between the nanomaterials and aquatic organisms in assessing  the fate, transport, and ecological impacts of nanomaterials. | [8] |
| MWCNT  >95% pure  10-30 nm x 10-30 µm  (Cheeptubes, Brattleboro, VT,USA)  Dispersed by stirring. | TEM + fractal analysis  BET  EDX  SEM  DLS  ZetaPALs (zeta size)  Absorbance 323 nm  Microscope | *Ceriodaphnia dubia*  Fresh water flea | Water bioassays  EPA/812/R-02/012  (in MHRW + 100 mg/L NOM)  Final concentrations:  MWCNTs 39.5-120.2 mg/L | Ingested MWCNTs were detected in large amounts in the gut and were depurated after 24 h only in the presence of algae. | [9] |

| **Particle** | **Characterization**  **method used** | **Model organism** | **Exposure condition, duration**  **and detection method** | **Conclusions** | **Ref** |
| --- | --- | --- | --- | --- | --- |
| DWCNT  (non-commercially available)  Sonicated and stirred, suspended in artificial seawater. | Elemental analysis (ref)  TEM | *Tigriopus japonicus*  Harpacticoid copepod | Copepod life cycle test | Copepod ingested DWCNTs and excreted them in faeces. DWCNTs were not evident in the cuticle or cuticle cells. | [10] |
| SWCNT  Electrophoretically purified. | TEM  Visible-light fluorescence  EDAX  AFM  Raman  Light and  confocal microscopy | *Amphiascus tenuiremis*  Meiobenthic estuarine copepod | 96-microplate life-cycle bioassay  (ASTM method E-2317-04)  Concentrations 0.58, 0.97, 1.6, and 10 mg/L | Copepod ingested SWCNTs as aggregates with algae and tightly packed agglomerates were excreted via feces. | [11] |
| SWCNT  MWCNT  (Not commercially available)  14C labelled  Sonicated in water for 30min. and tumbled in soil over night | TEM  TGA  Raman 785 nm | *Eisenia fetida*  Soil-dwelling earthworm | Uptake and Depuration experiments  Nominal concentration SWCNT 0.03 mg/g soil d.w. and MWCNT 0.03 and 0.3 g/kg soil d.w.  Exposure 1, 7, 14, 28 days (1d purging)  Depuration after 14 day exposure after 1, 2, 7 days. | CNTs are not readily absorbed and are eliminated with ingested soil. | [12] |
| SWCNTs  1-2 nm  MWCNTs  30-70 nm  (Not commercially available)  Mixed in pyrene spiked soil | TEM (ref)  TGA (ref)  Raman (ref) | *Eisenia fetida*  Soil-dwelling earthworm | Bioaccumulation and elimination  0.3, 3 g/kg soil  Exposure 1, 7, 14, 28 days  Depuration after 14 day exposure after 2, 6, 9 days unamended soil. | At 3.0 mg SWCNTs and MWNTs/g soil decreased pyrene bioaccumulation and elimination. NTs sorb nonpolar contaminants. Thus, presence of CNTs will decrease accumulation of HOC as “hard” carbons. | [13] |
| MWCNT  (Not commercially available)  14C labeled  Non-modified and Modified with PEI with +, - , 0 charge  Reconstituted in DI water, sonicated 30 min. and mixed in soil. | TEM  NMR (1H)  SEM  Biological oxidation + radioactivity  TGA  Zetasizer  X-ray photoelectron spectroscopy | *Eisenia fetida*  Soil-dwelling earthworm | Bioaccumulation and elimination  Exposure 2, 7, 14 and 28 days  Depuration after 14 day exposure after 2, 6, 9 days unamended soil. | Surface coating did not affect MWCNT uptake and CNTs were readily eliminated. | [14] |

| **Particle** | **Characterization**  **method used** | **Model organism** | **Exposure condition, duration**  **and detection method** | **Conclusions** | **Ref** |
| --- | --- | --- | --- | --- | --- |
| SWCNT and MWCNT  (not commercially available)  14C labelled  Dispersed by sonication, mixed to natural sediment. | TEM  TGA  Raman 785 nm  Biological oxidation + (Radioactive labeling) + scintillation counting  After:  Spectrophotometry | *Lumbriculus variegates*  Infaunal lugworm | Bioaccumulation and elimination  0.003 or 0.03 g SWCNT/kg dry sediment  0.037 or 0.37 g MWCNT/kg dry sediment  Exposure 7, 14, 28 days  rsure for 1, 2, 3 days. | CNT did not absorb in tissues and CNT readily eliminated after intake. Accumulation factors were factor lower to pyrene (PAH).  Will CNTs act as charcoal and limit hydrophobic organic material bioavailability?? | [15] |
| MWCNT  (not commercially available)  14C labelled  1)Purified  2) 3:1 (acid treated)  Dispersed by sonication, mixed to natural sediment. | TEM  SEM  TGA  XPS  Zetasizer  (Radioactive labeling) + scintillation counting | *Lumbriculus variegates*  Infaunal lugworm  *Eisenia foetida*  Earthworm | Exposure 1, 7, 14, 28 days, 6h depuration (after 14 days, 1, 2, 3 day purging).  0.37 g/kg sediment (d/w)  24h depuration  0.30 g/kg soil (d/w) | Purified and 3:1 MWCNTs had similar uptake and depuration by *L.variegates* and *E. Foetida.*  The oligochaetes excreted purified CNTs more consistently.  CNTs were not absorbed across the gut tissue or the dermis. | [16] |
| SWCNT  (cat no: 652512-250 MG, Sigma–Aldrich, UK)  96.32% pure C, 0.08%Al, 0.4% Cl, 2.9%Co, 0.29%S  1-2nm x 0.5-2.0µm  XRD 420m2/g  sonicated in MiliQ water and added to sediment/artificial sea water mixture. | Manufacturer – element analysis  TEM  BET  ICP-OES  coherent anti-Stokes Raman  scattering (CARS) microscopy in tissue | *Arenicola marina*  Marine infaunal lugworm | Exposure for 10 days  concentrations 0.003-0.03 g/kg | No uptake of SWCNT into tissues was observed, suggesting that these particles either remain in the sediment or pass through the gut and are excreted. | [17] |
| SWCNT  (Carbon Solutions Inc. Riverside, CA)  14C labeled  BET 233±3m2/g  Spiked alone or with HOC  Rolled in soil at 4C for week | BET  Raman  liquid scintillation fluid + direct counting of  gravimetric aliquots and combustion and quantitative trapping of evolved _14_CO_2_ | *Amphiascus tenuiremis*  Meiobentic copepod  *Streblospio benedicti*  polychaete | Bioaccumulation experiments  Exposure 14 days (followed by purging)  Nominal concentration 5 mg/g soil d.w. | A. tenuiremis and S. benedicti did not bioaccumulate SWCNTs. S. benedicti ingested soils containing CNTs, but particles were eliminated with fecal rods.  Addition of CNTs reduced bioaccumulation of HOCs in S.benedicti. | [18] |

| **Particle** | **Characterization**  **method used** | **Model organism** | **Exposure condition, duration**  **and detection method** | **Conclusions** | **Ref** |
| --- | --- | --- | --- | --- | --- |
| SWCNT  90% pure  (Sigma-Aldrich, St. Louis, MO, USA)  11 nm x 0.5-100 µm  Dispersed by 30 min. Stirring in filtered tap water. | EDX - %C  Inverted microscopy  SEM | *Danio rerio*  zebrafish embryos | 20h exposure from 4hpf | Chorion acts as a strong protective barrier and prevents passage of NTs through the pores.  NTs adhered to the chorion. | [19] |
| MWCNT  (Nanostructured and Amorphous Materials)  Carboxylised and FITC-BSA-conjugated by amidation.  Fluorescent-labelled. | TGA  Radioactive labelling  S-TEM  SEM  TEM  AFM  After:  TEM  Inverted microscopy | *Danio rerio*  zebrafish embryos | Microinjection at 1-cell stage embryos and at 72 hours post fertilization  Concentration 2 ng/embryo | In blastoderm, cells CNT remained through proliferation, and were distinctively excluded from the yolk cell.  In circulation system, CNT moved in embryonic compartments and were cleaned out by the body at 96 hours after the loading. | [20] |
| MWCNT  (Graphistrength C100, France)  DWCN  97.7% pure  (non-commercially available)  BET 800, 900 m^2^/g  0.7-2.2 nm  Suspended in reconstituted water. | 2008/2009:  Element analysis  BET  Raman  SEM  TEM  XRD  After:  Microscope (2008)  TEM (2008)  Raman (2008, 2009)  2010:  TEM  BET  Raman  After:  Raman 488 nm | *Xenopus laevis*  amphibian larvae | 12-day exposure from developmental stage 50  Nominal concentrations 0.1 to 50 or 100 mg/L | CNT were detected in the lumen of the intestine, but did not cross the intestinal cells. Toxicity was observed to physical blockage of gills and digestive tract. | [21-24] |

| **Particle** | **Characterization**  **method used** | **Model organism** | **Exposure condition, duration**  **and detection method** | **Conclusions** | **Ref** |
| --- | --- | --- | --- | --- | --- |
| SWCNT  (HiPco)  1 nm x 1600 nm  Suspension in Bovine serum albumin was sonicated, centrifuged, decanted and spiked in dry yeast. | NIR | *Drosophila melanogaster*  fruit fly | Concentration ~10 mg/L in food | Drosofila ingested CNTs and they were mostly excreted. Small fraction translocated into hemolymph and from here to the primitive brain and less to other tissues. | [25] |
| SWCNT  70% pure  (Ni:Ycatalyst, CSI, Riverside, CA)  MWCNT (MER, Tuscon, AZ)  Sonicated in solvent. | Manufacturer data  TEM  FE-SEM | *Drosophila melanogaster*  fruit fly | Toxicity test (egg to adult)  CNT stirred in the larval food, gels with dose 0.1 and 1.0g/kg | Ingested by larvae and visible also in developing flies. | [26] |
| SWCNT  90% pure  carboxylated  ∅1.4 nm  Labeled ^125^I | Raman  TEM  Micro-FTIR  Hydrodynamic radius  Spectroscopy | KM mice (male) | Oral gavage  (3.52x10^6^ cmp/mL, 15µg/mL)  6h, 3d, 6d, 11d, 18d post injection | High concentrations in stomach, kidney, lungs, bone 3h.  Low concentrations in brain, heart and muscle 3h. | [27] |
| MWCNT  >95% pure  Ni, Fe, Co impurities <1%  ∅10-20 nm x 600nm  ^14^C-Tarine functionalized | TEM  TGA  ICP-MS  XPS  Elemental analysis | KungMing mice (male) | Oral gavage  10µg  12-h post exposure | CNT quickly passed through the digestive system and 12-h post exposure 74% were excreted in feces. | [28] |
| MWCNT  (Sigma)  >95% pure  5-40/40-70nm x 0.5-2 µm  aggregate ∅ 240nm  Suspended in NOM (100mg/L), sonicated 30min | Zetasizer | *Oryza saliva*  Rice plant | Seed MWCNT-NOM pretreatment and 6m growth.  Nominal concentrations 2.5-800mg/L | Few aggregates were observed in vascular system and fewer in the plant tissues. | [29] |

| **Particle** | **Characterization**  **method used** | **Model organism** | **Exposure condition, duration**  **and detection method** | **Conclusions** | **Ref** |
| --- | --- | --- | --- | --- | --- |
| SWCNT  (Carbon Solutions, Riverside, CA)  P3-SWCNT non-functionalyzed  80-90% pure  5-10% metal content by wt  P8-SWCNT functionalyzed  80-90% pure  3% metal content by wt  Dispersed in DI water and sonicated for 30 min and 90 min | SEM  After:  SEM | *Cucumis sativus*  Cucumber | Seedling exposure for 48 h in hydroponic conditions. | No CNT uptake, only on root surfaces. No differences between CNTs. | [30] |
| MWCNT  (Arry International, GE)  60% pure  ~30 nm  O-MWCNT (oxidized)  ~20 nm  Dispersed in DI and sonicated | FTIR  FESEM  XRD  Plants:  TGA  Conductivity  UV/VIS Spectrometer | Mustard plant | Time-50 Germination  Nominal concentration  MWCNT 23, 46 mg/L  O-MWCNT 2.3, 6.9 mg/L | MWCNT and O-MWCNT penetrated seed coat and root tissue. | [31] |
| MWCNT  (not commercially available)  10-35nm x 6µm  98.5% pure  carboxylized  Dispersed in Marushige and Skoog growth medium. | PT  PA  Optical microscope Raman  TEM | Tomato plant | Plant growth  Nominal concentration 50 mg/L | CNT were detected in root, leaves, and fruits. | [32] |

| **Particle** | **Characterization**  **method used** | **Model organism** | **Exposure condition, duration**  **and detection method** | **Conclusions** | **Ref** |
| --- | --- | --- | --- | --- | --- |
| MWCNT  (not commercially available)  98% pure  Dispersed in Marushige and Skoog growth medium. | TEM  TGA  Raman | Tomato plant | 2-day seed incubation  Nominal concentrations 40 mg/L | CNT entered the seeds. | [33] |
| MWCNT  (Sigma-Aldrich)  110-170nm x 9µm  Excitation 710nm  Emission 360-530nm  Sonicated for 12h in suspension | TPEM  (two-photon excitation  Microscopy) | Wheat | Root growth 96-h intervals  Nominal concentrations 100 mg/L | CNT pierced wheat roots, but did not fully enter or encapsulate in cells. | [34] |

MICROBIAL TOXICITY

| **Particle** | **Characterization**  **method used** | **Model organism** | **Exposure condition, duration**  **and detection method** | **Conclusions** | **Ref** |
| --- | --- | --- | --- | --- | --- |
| SWCNT  (not commercially available)  90% pure  0.8 wt% Co  0.9 nm  Suspended in 0.9%NaCl | Raman 785 nm  TEM  Element analysis  TGA  After:  SEM | *Escherichia coli K12*  Gram-negative bacteria | Bacterial toxicity  In suspension 1-5mg/L  Deposited on CNT-coated filter | Both in suspension and on filter contact with pristine SWCNT aggregates induced toxicity.  Toxicity ↑ with time. | [35] |
| SWCNT  (Stanford Materials; SWCNT-90)  1.2 nm diameter  10-20 nm  6% w/w metals  Sonicated for 30 min., | Raman 532 / 785 nm  TEM  Element analysis  TGA | *Escherichia coli K12*  Gram-negative bacteria | Biofilm Development Assay  0.5 – 500 mg/L  48 incubation at 30˚C | Concentrations >300 mg/L inhibited cell growth and biofilm formation. Lower concentrations induced bacterial growth due to bacterial utilization of dead cells.  Mature biofims were less sensitive than developing biofilms.  Surfaces coated with SWCNTs have 10x<biofilm development. | [36] |
| SWCNT  0.81wt% metal catalysts  0.9nm x 2µm  MWCNT  0.62wt% metal catalysts  30nm x 70µm | Raman 785 nm  TEM  Element analysis  TGA  After:  SEM | *Escherichia coli K12*  Gram-negative bacteria | Bacterial toxicity  In suspension 5mg/L  Deposited on CNT-coated filter | SWCNT in suspension or on filter induced more cell death, less metabolic activity, more plasmid DNA, and more stress related gene products than MWCNTs.  Bacterial death was related to CNT contact and membrane damage. | [37] |

| **Particle** | **Characterization**  **method used** | **Model organism** | **Exposure condition, duration**  **and detection method** | **Conclusions** | **Ref** |
| --- | --- | --- | --- | --- | --- |
| MWCNT  (Yadkinville, NC)  1) As prepared AP-MWCNT  17 nm x 91 µm  2) Dry oxidized DO-MWCNT at 350˚C for 6 h  20 nm x 84 µm  3) Acid treated AT- MWCNT 10 M HCl at 70˚C for 18 h  17 nm x 77 µm  4) Functionalized f-MWCNT by sonication in acid mixture for 1 h at 22˚C  19 nm x 4.1 µm  5) Annealed for structural imperfections AN-MWCNT  21 nm x 82 µm  6) Short s-MWCNT  35 nm x 2.3 µm | Raman 532 nm  TEM  SEM  TGA  EDX | *Escherichia coli K12*  Gram-negative bacteria | Bacterial toxicity assays  (cell membrane integrity and cell metabolic activity) | Pre-treatment, purification and functionalization modifications of MWCNTs (altered physicochemical properties) have modest effect on bacterial toxicity.  Uncapped, de-bundled, short and dispersed nanoparticles (f-MWCNT and s-MWCNT) were more cytotoxic. | [38] |
| SWCNT  (not commercially available)  Dispersed by sonication for 1h.  1) 0.9% NaCl  2) 0.1% wt Tween-20 + 0.9% NaCl | Raman  TGA / DTA  Photoluminescence  UV-vis-IR absorption  AFM  SEM  TEM | *Escherichia coli*  *Pseudomonas aeruginosa*  Gram negative bacteria  *Bacillus subtilis*  *Staphylococcus aureus*  Gram-positive bacteria | Baterial Activity  Concentration 5, 10, 20, 40, 80 mg/L for 2h.  Membrane integrity  Concentration 5 mg/L for 2h.  OD growth | Individually dispersed SWCNT (2) were more toxic than aggregated SWCNT (1).  Gram-positive bacteria were more sensitive to SWCNTs than gram-negative bacteria.  Effects due to membrane damage by physical punctures by SWCNTs and some oxidative stress. | [39] |

| **Particle** | **Characterization**  **method used** | **Model organism** | **Exposure condition, duration**  **and detection method** | **Conclusions** | **Ref** |
| --- | --- | --- | --- | --- | --- |
| SWCNT  ~1.5nm x 10µm  MWCNT  ~15-30nm x 1-5 µm  (NanoLab, Inc., Newton, USA)  With functional groups:  -OH  -COOH  -NH2  Suspended in DI, or 0.9%NaCl, or 0.1M PBS, or BHI broth | Manufacturer data  After:  Fluorescence microscopy  SEM | *Salmonella typhimurium*  Gram-negative rod bacteria  *Bacillus subtilis*  Gram-positive rod bacteria  *Staphylococcus aureus*  Gram-positive sphare bacteria | Bacterial toxicity  Concentrations 100-500mg/L | At 100µg/mL SWCNT –OH & -COOH aggregates in DI and NaCl were antimicrobial to all bacteria (wrap around bacteria with needle-like action). Toxicity ↑ with time and concentration. No effect at same concentration in PBS or BHI broth (less ionic strength).  SWCNT-NH2 were toxic at 500mg/L (did not aggregate).  MWCNT –OH, -COOH, -NH2 NOEC 500µg/mL in all media (settle on bacterial surface with loose contact). | [40] |
| SWCNT  (NanoLab, Inc., Newton, USA)  95.93% pure  4.07% (Na, Al, Si, S, Fe)  -OH functionalized  1-1.5nm x <1µm  1-1.5nm x 1-5µm  1-1.5nm x ~5µm  Suspendet in DI water | Fluorescence Imaging  SEM  TEM | *Salmonella typhimurium*  Gram-negative rod bacteria | Bacterial toxicity  Concentration 50, 100, 150, 200mg/L  Exposure 1-5h | Longer CNTs aggregated with bacteria and induced toxicity ↑ with time and concentration.  Short CNTs aggregated alone, reducing toxicity. | [41] |
| SWNT  >95% pure (>5% metallic  ~30% metallic  >95% metalic  (not commercially available) | TEM  Vis-NIR  Raman  After:  SEM | *Escherichia coli K12*  Gram-negative bacteria | Bacterial toxicity assays  (cells suspended in 1 mg/L or cells deposited on SWNT-coated filter) | SWNT electronic structure affected microbial activity. Bacterial toxicity increased with metallic content and occurred shortly after contact. Toxicity was mediated by glutathione oxidation. | [42] |

| **Particle** | **Characterization**  **method used** | **Model organism** | **Exposure condition, duration**  **and detection method** | **Conclusions** | **Ref** |
| --- | --- | --- | --- | --- | --- |
| SWCNT  99% pure  (Stanford Materials, Aliso Viejo, CA)  1.2 nm x 17.8 µm  MWCNT  99% pure  NanoTechLabs Inc., Yadkinville, NC)  17.4 nm x 77 µm  Purified with HCl | Raman 532 nm  TEM  SEM  TGA  XPS (element analysis) | *Escherichia coli*  *Pseudomonas aeruginosa*  Gram-negative bacteria  *Bacillus subtilis*  *Staphylococcus epidermidis*  Gram-positive bacteria  Microbial community from river water  Microbial community from wastewater effluent | Bacterial toxicity assays  (cell membrane integrity) | CNts were cytotoxic to the tested microbial models (SWCNT>MWCNT) and toxicity increased with exposure time.  Adding natural organic matter did not affect the toxicity of SWCNTs, despite reduced deposition and attachment of bacteria on the CNT surface. | [43] |
| SWCNT  MWCNT  (not commercially available)  Treated with HNO3, dialyzed, dispersed in ultra-pure water and diluted in 3% NaCl | After:  TEM | Luminescent bacteria | Acute Toxicity Luminescent bacteria Test Chinese EPA GB/T15441-1995 | EC50  SWCNT (1-2nm) 33 mg/L  MWCNT (≤8 nm) 50 mg/L  MWCNT (20-30 nm) 54 mg/L  MWCNT (≥ 50 nm) 84 mg/L | [44] |
| SWCNT  (Southwest Nanotechnologies, Inc., USA) 90% pure  1nm x 1µm  1125.3m^2^/g | TGA  BET | Soil bacteria | Enzyme activity (2h, 3d, 7d, 15d, 23d)  Bacterial growth (32d)  Concentration 30, 100, 300, 600, 1000 mg/kg soil | Activity of tested enzymes was repressed at 1000 mg/kg.  Microbial mass lowered LOEC 300mg/kg. | [45] |
| **Particle** | **Characterization**  **method used** | **Model organism** | **Exposure condition, duration**  **and detection method** | **Conclusions** | **Ref** |
| MWCNT  Sigma  >90% pure  1) 10-15nm x 0.1-10µm  2) 110-170nm x 5-9µm Supended in 1% BSA in Tryptic soy broth soybean-casein medium, shaken. | Manufacturer data | *Pseudomonas fluorescens*  Gram negative bacteria | Assessment of metabolic function  Concentrations 0.0025, 0.005, 0.01, 0.015mg/L | Dose related functional changes related to membrane disruption attributed by oxidative damage.  NOEC 0.015mg/l cytotoxicity  Short>Long | [46] |
| MWCNT  (CEA)  44nmx1.5 µm  SSA42 m2/g  1) Raw (Fe-coated)  4.24wt%Fe  2) Pure (heat-purified)  0.08wt%Fe  Dispersed in ultrapure sterile water with solvent gum Arabic (0.25 wt.%), sonicated. | BET (SSA)  TEM  After:  Zeta potential  Point of Zero Charge  XRD | *Escherichia coli*  Gram-negative bacteria  *Cupriavidus metallidurans*  Gram-negative bacteria | Bacterial growth | 1) Bactericidal to *E. coli* at 100 mg/L, *C. metallidurans* was unaffected  2) Bactericidal to *E. coli* at 100 mg/L, *C. metallidurans* was unaffected. | [47] |
| MWCNT  96% pure  (Hanwha Nanotech, rep. of Korea)  15 nm x 10-20 µm  Treated with 23% HNO3 at 80˚C, dispersed in DI water and stirred 2h, washed and dryed.  Dispersed in DI water, sonicate 5 min., mixed in soil. | BET  TGA  Raman  TEM  Metal analysis | Soil bacteria | Enzyme activity and bacterial growth  Concentration 50, 500, 5000 mg/kg soil  1) Sandy loam soil  2) Loamy sand soil | Activity of tested enzymes was repressed at 5000 mg/kg in both soil types.  Microbial mass was lowered after 20 day incubation at 5000 mg/kg in both soil types. | [48] |

| **Particle** | **Characterization**  **method used** | **Model organism** | **Exposure condition, duration**  **and detection method** | **Conclusions** | **Ref** |
| --- | --- | --- | --- | --- | --- |
| MWCNT  (Trading Echo-Nanobio Co.)  40-60nm x 300-600nm  140-300 g/dm^3^  1) Raw >80%  2) MWCNT-COOH >90%  Suspended in Di and shaken 36days. | Manufacturer data  After:  Fluorosecnce microscopy  SEM | *Paecilomyces fumosoroseus*  Entomopathogenic fungi | Fungal growth  CNT-conidium contact for 1 to 865-h, followed by mycelium growth fase.  Concentration 0.2mg/L | No effect on growth.  Sporification affected without time-response pattern. | [40] |

| **AQUATIC TOXICITY** |  |  |  |  |  |
| --- | --- | --- | --- | --- | --- |
| **Particle** | **Characterization**  **method used** | **Model organism** | **Exposure condition, duration**  **and detection method** | **Conclusions** | **Ref** |
| MWCNTs  (Cheaptubes, VT, USA)  95% pure  5-10 nm (20-30 nm) x 10-30 µm  Mixed in soil for 6h | Manufacturer data | Bentic macroinvertebrate community and aquatic macrophates | Community recolonization experiment  (sediment with MWNTs was placed in donor system for 3m)  Concentrations 0.002, 0.02, 0.2, 2 g/kg d.w. (Env. Relevant) | With increased MWCNT concentration increased the number organisms.  No effect on biodiversity.  Some effects on community level. | [49] |
| SWCNT  (Sigma-Aldrich, no. 519308)  50-70% pure  1.2-1.5 nm x 2-5 µm  1) Suspended in water (ASTM grade no.1) and mixed (12 RPM, 24 h)  2) Filtered through 0.22 µm filter  3) In some cases CRM sediment (NWRI, 2000) was added | Manufacturer data | *Vibrio fisheri*  Marine bacteria  11 bacterial species  *Pseudokirchneriella subcapitata*  Fresh water algae  Photosynthetic enzyme complexes  *Thamnocephalus platyurus*  Fresh water crustacean  *Hydra attenuata*  Frash water dihydroid  *Oncorhynchus mykiss*  Rainbow trout  *Vibrio fisheri*  Marine bacteria  Photosynthetic enzyme complexes | Liquid phase assays:  Microtox test  MARA assay  Algal microplate assay  Luminotox Assay  ThamnoToxkit assay  Cnidrian test  Fish cell test (primary hepatocytes)  Solid phase assays:  Microtox test  Luminotox Assay  Max. concentration 185 mg/L, pH 6.8 | Categorized as:  Toxic (1-10 mg/L) for Cnidrian test and Algal microplate assay.  Not toxic (>100 mg/L) for remaining assays.  Including CRM sediment for solid phase assays did not affect the results. | [50] |

| **Particle** | **Characterization**  **method used** | **Model organism** | **Exposure condition, duration**  **and detection method** | **Conclusions** | **Ref** |
| --- | --- | --- | --- | --- | --- |
| SWCNT  (Rice HPR 145.1)  Suspended in gum Arabic (GA)  1) with 0.023% (v/v) GA  2) with 0.046% (v/v) GA  Mixed for 1.5h, sonicated 10 min, | AFM  TGA/SDTA  ICP-AES  After:  Vis-NIR  Raman  TEM | *Pseudokirchneriella subcapitata*  Fresh water algae | a) 96-h algal bioassay (1003.0 USEPA)  Nominal concentration 0.01-0.5mg/L  b) Long term (2week) growth study  Nominal concentration 0.5mg/L and GA 0.023%. | 1a) Growth inhibition LOEC 0.25mg/L  2a) Growth inhibition NOEC 0.5mg/L  1+2b) P. subcapitata recovered from the initial toxicity. | [51] |
| CNT  (EPFL Lausanne, SW)  99% pure  5-15nm x 2-5 µm  1) Pristine  2) Oxidized  Dispersed in Milli-Q water with NOM, sonicated, added to algal medium. | ICP-MS  TEM  SEM  XPS  Zetasizer  DLS | *Chorella vulgaris*  Green algae | Algal growth test 96-h (OECD 201)  Photosynthetic activity  Final concentrations  1) 5.5 ± 1.8 mg/L  2) 6.9 ± 1.9 mg/L | 1) Growth inhibition (due to agglomeration)  EC(50) 1.8-24 mg/L  LOEC 0.5-13mg/L  NOEC 0.04-3.0mg/L  2) Growth inhibition (due to agglomeration)  EC(50) 2.5-40 mg/L  LOEC 0.5-13mg/L  NOEC 0.2-3.0mg/L  No effect on photosynthetic activity | [52] |
|  |  | *Pseudokirchneriella subcapitata*  Fresh water algae | Algal growth test 96-h (OECD 201)  Photosynthetic activity  Final concentrations  1) 5.5 ± 1.8 mg/L  2) 6.9 ± 1.9 mg/L | 1) Growth inhibition (due to agglomeration)  EC(50) 20-36 mg/L  LOEC 5.5-13mg/L  NOEC 1.3-3.0mg/L  No effect on photosynthetic activity |  |
| MWCNT  (Shenzhen Nanotech Port Co., Ltd., China),  Outer ∅ 10, 20-40, 60-100nm  Purification in HCl or algal cell culture medium  Bath sonication in medium (100W, 40kHz, 25°C, 15min) | TEM  SEM  BET | *Chorella vulgaris*  Green algae | 96 h growth inhibition, exposure with and without illumination, TBARS assay, H_2_DCFDA assay  Concentrations 0-100 mg/l | MWCNT significantly inhibited algal growth, contribution of metal catalyst and binding of nutrients to growth inhibition was negligible  Tox. Was mainly explained by combined effects of oxidative stress, agglomeration and physical interactions, and shading effects | [53] |

| **Particle** | **Characterization**  **method used** | **Model organism** | **Exposure condition, duration**  **and detection method** | **Conclusions** | **Ref** |
| --- | --- | --- | --- | --- | --- |
| SWCNT  (Hippo tube, Carbon Nanotechnologies)  Refluxed in HNO3  2-10 nm x <500 nm  Mixed in a complex ciliate growth medium, proteose peptone extract. | AFM  SEM  EDAX  After:  SEM | *Tetrahymena thermophila*  Unicellular ciliated protozoan | Chronic toxicity test (72-h)  Bacterivory bioassays  Concentrations 0-17.2mg/L | Mobility loss LOEC 1.6mg/L  Cell death LOEC 1.6mg/L  Bacterivory LOEC 3.6mg/L  Viability LOEC 6.8mg/L | [1] |
| MWCNT  (Cheap Tubes Inc, MFG#Mw20-30nm95)  20-30nm x 50 µm  95% pure (5%O2, 0.1% Co)  1) Oxidised and dissolved in artificial seawater.  2) Filtered 0.2 µm filtration. | SEM  EDX  Efter:  SEM  Coulter counter  DLS | *Dunaliella tertiolecta*  marine green algae | Chronic toxicity test  Concentration 0.1, 0.5, 1, 2.5, 5, and 10mg/L | 1) Growth inhibition EC(50) 0.82mg/L.  Oxidative stress LOEC 10 mg/L  Photosynthesis inhibition LOEC 10mg/L.  2) Growth inhibition NOEC 10mg/L | [54] |
| DWCNT  (non-commercially available)  1) Sonicated, suspended in artificial seawater.  2) Stirred, suspended in artificial seawater. | Elemental analysis  TEM | *Thalassiosira pseudonana*  marine diatom | 96-h algal bioassay | 1) Growth inhibition LOEC 0.1mg/L EC(50) 1.86 mg/L  2) Growth inhibition LOEC 0.1mg/L EC(50) 22.7mg/L | [10] |

| **Particle** | **Characterization**  **method used** | **Model organism** | **Exposure condition, duration**  **and detection method** | **Conclusions** | **Ref** |
| --- | --- | --- | --- | --- | --- |
| MWCNT  (method by Shenzhen Nanotech Port Co., Ltd., China)  95% pure (0.1%Ni, 0.2%Fe, 0.4%N)  60-100 nm x 600 nm  Dispersed in pond water medium and sonicated | Raman 632.81 nm  SEM  AFM  TEM  ICP-MS  FTIR  After:  Mikroskop 90X  Fluorescens mikroskop  EM | *Stylonychia mylilus*  Unicellular protozoan | 5 day toxicity study  Concentrations 0.1-200 mg/L | Growth inhibition LOEC 1.0 mg/L (70%). | [2] |
| SWCNT  >60% pure  2 nmx5-15 µm  (Shenzhen nanotech Port Co., China)  Suspended in reconstituted water, shaken at room temperature. | Manufacturer data  After:  Microscopy | *Daphnia magna*  Fresh water flea | 48-hour acute immobilization test  (modified OECD Guideline 202)  Concentrations ranging from 0.1-100mg/L  (not fed during test) | Immobilization EC(50) 1.3mg/L.  Morality LC(50) 2.4mg/L. | [3] |
| SWCNT-LPC  (Carbon Nanotechnologies)  2 nm x 0.3 nm  Coated with lysophosphatidylchlorine by sonication 30 min at 24˚C  SWCNT-LPC + Cu mixture | Raman  TEM | *Daphnia magna*  Fresh water flea | Acute (48 and 96 h) water bioassay  EPA/600/4-90/027F  Nominal concentrations SWCNT-LPC:  1-10mg/L (48h) and 0.1-1mg/L (96h)  0.5 and 1 toxic unit (TU)  (fed during test) | Mortality LC(50) 6mg/L (48h)  Mortality LC(50) 0.05mg/L (96h)  Toxicity was additive.  LPS-SWCNT enhanced uptake and toxicity of Cu. | [55] |
| HiPCO SWCNTs  (Carbon Nanotechnologies Inc., TX)  85% pure  1.2 nm diameter  Solubilized by LPC | Spectrophotometric analysis  TEM  After:  Micro-Raman 514.5 nm  Fluorescent microscopy | *Daphnia magna*  Fresh water flea | 96-hour acute toxicity test  Nominal concentrations 0, 2.5, 5, 10, 20mg/L | Morality NOEC 5mg/L  Mortality (20%) LOEC 10mg/L  Toxicity was likely due to adhered particles on external surface of daphnids.  Daphia ingested LPC, reduced CNT solubility | [5] |

| **Particle** | **Characterization**  **method used** | **Model organism** | **Exposure condition, duration**  **and detection method** | **Conclusions** | **Ref** |
| --- | --- | --- | --- | --- | --- |
| SWCNT  (NanoAmor, TX)  >95% pure  20-30nm x 0.5-2 µm (declared)  Dispersed in reconstituted water with 15mg/L NOM, sonicated 30 min and diluted.  Media pH 6, 7, or 8  pH 6: aggregate ∅ 9.2 µm SEM, 129nm DLS, -22 zetapotential  pH 7: aggregate ∅ 6.5 µm SEM, 149nm DLS, -23 zetapotential  pH 7: aggregate ∅ 2.3 µm SEM, 142nm DLS, -26 zetapotential | SEM  DLS  Zeta | *Daphnia magna*  Fresh water flea | 96-hour acute toxicity test  Reproduction (3^rd^ brood)  Nominal concentrations  pH 7: 0.5, 1, 2, 4 mg/L  pH6 or 8: 1, 2, 4, 10 mg/L | Mortality NOEC 4 or 10mg/L  Growth LOEC 4.8mg/L  Reproduction LOEC 0.24mg/L (56%) (pH7) | [56] |
|  | SEM  DLS  Zeta | *Ceriodaphnia dubia*  Fresh water flea | 96-hour acute toxicity test  Reproduction (3^rd^ brood)  Nominal concentrations  pH7: 0.5, 1, 2, 4 mg/L  pH6 or 8: 1, 2, 4, 10 mg/L | Mortality and growth NOEC 4 or 10 mg/L  Reproduction LOEC 0.48mg/L (~78%, pH6;8) |  |
| MWCNT  (not commercially available)  14C labelled  1) HNO3/H2SO4 (3:1) treated  2) Grafted with PEI (polyethylenemine) | NMR (1H)  Zeta potential  TEM  Biological oxidation and measured radioactivity | *Daphnia magna*  Fresh water flea | Immobilization test (24h and 48h)  Concentrations 0-40mg/L | 1) Immobilization EC(50) 24h ~25mg/L  Immobilization EC(50) 48h 12.7mg/L  2) Immobilization EC(50) 24h ~17mg/L  Immobilization EC(50) 48h ~9mg/L  Increased toxicity due to PEI was not due to surface charges, but rather due to the size. | [7] |
| MWCNT  >98% pure  10-20 nm x 5-15 µm  (Shenzhen nanotech Port Co., China)  Suspended in reconstituted water, shaken at room temperature. | Manufacturer data  After:  Microscopy | *Daphnia magna*  Fresh water flea | 48-hour acute immobilization test  (modified OECD Guideline 202)  Concentrations ranging from 0.1-100mg/L  (not fed during test) | Immobilization EC(50) 8.7mg/L.  Morality LC(50) 22.8mg/L. | [3] |

| **Particle** | **Characterization**  **method used** | **Model organism** | **Exposure condition, duration**  **and detection method** | **Conclusions** | **Ref** |
| --- | --- | --- | --- | --- | --- |
| MWCNT  (Hanhwa Nanotech, Korea)  95% pure  10-15 nmx 10-20 µm (declared)  Suspended in NOM solution, sonicated 5-7 min at 30W, resuspended and sonicated (total 4x). | Manufacturer data  Efter:  TEM  EDS  FIT-IR | *Daphnia magna*  Fresh water flea | Acute (48 and 96 h) water bioassay  EPA/600/4-90/027F  Nominal concentrations 0, 1.25, 2.5, 10, 20mg/L MWCNTs with 1mg/L Cu  Combinations of MWCNTs, Cu and NOM mixtures | 48h mortality NOEC 20mg/L  96h mortality LC(50) 2.48mg/L  Bioavailability of Cu was enhanced by the presence of MWCNTs interacting with NOM (membrane disruption by MWCNTs). | [57] |
| MWCNT  >95% pure  (not commercially available)  25 nm x 50 µm  Suspended in 3 different types NOM suspensions (0.2 µm filtered), sonicated for 25 min, diluted and sonicated for 1h, after 24h settled sediment was removed. | DLS  Zetasizer  SEM  IR  After:  TEM | *Daphnia magna*  Fresh water flea | Acute 96h static renewal bioassays  (USEPA/600/4-90/027F, USEPA/600/4-91/002) | LC(50) 2-4mg/L (depending on NOM)  Growth LOEC 0.25mg/L | [4] |
|  | DLS  Zetasizer  SEM  IR  After:  TEM | *Ceriodaphnia dubia*  Fresh water flea | Acute 96h static renewal bioassays  Chronic 7 day toxicity | LC >1mg/L  Growth LOEC 0.2mg/L  Reproduction LOEC 0.125mg/L |  |

| **Particle** | **Characterization**  **method used** | **Model organism** | **Exposure condition, duration**  **and detection method** | **Conclusions** | **Ref** |
| --- | --- | --- | --- | --- | --- |
| MWCNT  1) 14.1 nm  2) 34.6 nm  3) 59.2 nm  Dispersed in water  a) Ozone 0.14g/min in US-bath 24h  b) Stirred 1h and US-probe 60 min  c) Stirred 24h | TEM  XRD  TOC  DLS  Microscope  Zeta potential | *Ceriodaphnia dubia*  Fresh water flea | Acute 24h mortality test (USEPA 1002)  3-brood reproduction test  Growth assay 48h  Concentrations 1-200mg/L | Acute 24h mortality test  1-3a) LC(50) 100mg/L  1b) LC(50) 8mg/L  2b) LC(50) 7 mg/L  3b) LC(50) 2mg/L  1c) LC(50) 17mg/L  2c) LC(50) 8mg/L  3c)LC(50) 20mg/L  3-brood reproduction test  3a) EC(50) 17mg/L  3b) EC(50) 4mg/L  Growth assay  a) LOEC 5mg/L  b) LOEC 2mg/L (NOEC 1mg/L) | [8] |
| MWCNT  >95% pure  10-30 nm x 10-30 µm  (Cheeptubes, Brattleboro, VT,USA)  1) Raw rMWCNT (150 m^2^/g)  D: 216 nm, Z: -24 mV  2) MWNT-OH (115 m^2^/g)  D: 183 nm, Z: -22 mV  3) MWCNT-COOH  D: 185 nm, Z: -23 mV  4) rMWCNT oxidized by 30% HNO3 at 80˚C for 6 h (140 m^2^/g)  Dispersed by stirring. | TEM + fractal analysis  BET  EDX  SEM  DLS  ZetaPALs (zeta size)  Absorbance 323 nm  Microscope | *Ceriodaphnia dubia*  Fresh water flea | Acute (48 h) water bioassay  EPA/812/R/02/012  (in MHRW+100 mg/L NOM)  Final concentrations:  rMWCNT 39.5mg/l  MWCNT-OH 120.2mg/L  MWCNT-COOH 88.9 mg/L | 1) Mortality LOEC 39.5 mg/L  48-h EC50 50.9 mg/L  2) Mortality NOEC 120.2mg/L  3) Mortality NOEC 88.9mg/L | [9] |
|  |  | *Leptocheirus plumulosus*  Marine amphipod | Acute (10 d) whole-sediment bioassay in 20‰ sea water  EPA/600/R-94/025  Nominal concentrations:  4, 10, 33, 99, 30 g/kg sed. | Mortality LOEC 99g/kg  LC50 68 g/kg |  |
|  |  | *Hyalella azteca*  Freshwater crustacean | Acute (10 d) whole-sediment bioassay in dechlor. tap water  EPA/600/R-99/064  Nominal concentrations:  3, 9, 29, 87, 264g/kg sed. | Mortality LOEC 264g/kg  LC50 >264g/kg |  |

| **Particle** | **Characterization**  **method used** | **Model organism** | **Exposure condition, duration**  **and detection method** | **Conclusions** | **Ref** |
| --- | --- | --- | --- | --- | --- |
| MWCNT  >95% pure  10-30 nm x 10-30 µm  (Cheeptubes, Brattleboro, VT,USA)  1) Raw rMWCNT  2) MWNT-OH  3) MWCNT-COOH  4) MWCNT-C8  5) MWCNT-NH2  Dispersed by:  a) stirring 30 min.  b) sonication for 15 min.  c) sonication 15 min. in NOM. | TEM  Surface area + diameter  ZetaPAL  DLS | *Ceriodaphnia dubia*  Fresh water flea | Acute (48 h) water bioassay  EPA/812/R/02/012  (stirred in MHRW + 100 mg/L NOM) | 1) Mortality LOEC 16mg/L  2) Mortality LOEC 48mg/L  3) Mortality LOEC 48mg/L  4) Mortality LOEC 15mg/L  5) Mortality LOEC 2mg/L  4a) LC50 17mg/L  4b) LC50 21mg/L | [58] |
|  |  | *Leptocheirus plumulosus*  Marine amphipod | Acute (10 d) whole-sediment bioassay  EPA/600/R-94/025 | a) Mortality LOEC 30g/kg  b) Mortality LOEC 30g/kg  c) Mortality LOEC 30g/kg |  |
|  |  | *Hyalella azteca*  Freshwater crustacean | Acute (10 d) whole-sediment bioassay  EPA/600/R-99/064 | a) Mortality LOEC >300g/kg  b) Mortality LOEC 300g/kg  c) Mortality LOEC >300g/kg |  |
| DWCNT  (non-commercially available)  1) Sonicated, suspended in artificial seawater.  2) Stirred, suspended in artificial seawater. | Elemental analysis  TEM | *Tigriopus japonicus*  Harpacticoid copepod | Copepod life cycle test | 1)  Larval mortality LOEC 30 mg/L  Population growth inhibition LOEC 0.1mg/L  2)  Larval mortality LOEC 100mg/L  Population growth inhibition LOEC 10mg/L | [10] |
| SWCNT  1) “As prepared” (AP).  2) As electrophoretically purified.  3) As the fluorescent fraction nanocarbon synthetic byproducts  dispersed in sea water. | TEM  Visible-light fluorescence  EDAX  AFM  Raman  Light and  confocal microscopy | *Amphiascus tenuiremis*  estuarine copepod | 96-microplate life-cycle bioassay  (ASTM method E-2317-04)  (28-35 d)  Concentrations 0.58, 0.97, 1.6, and 10mg/L | 1) Life-cycle mortality LOEC 10mg/L  Fertilization reduction LOEC 10mg/L  Molting reduction LOEC 10mg/L.  2) Life-cycle mortality, development or reproduction NOEC 10mg/L  3) Life-cycle mortality LOEC 10mg/L  Molting reduction LOEC 0.58mg/L | [11] |

| **Particle** | **Characterization**  **method used** | **Model organism** | **Exposure condition, duration**  **and detection method** | **Conclusions** | **Ref** |
| --- | --- | --- | --- | --- | --- |
| SWCNT  (90%, cat no: 652512-250 MG, Sigma–Aldrich, UK)  96.32% pure C, 0.08%Al, 0.4% Cl, 2.9%Co, 0.29%S  1-2nm x 0.5-2.0µm  XRD 420m2/g  Sonicated in MiliQ water, added to sediment/artificial sea water. | Manufacturer – element analysis  TEM  BET  ICP-OES  coherent anti-Stokes Raman  scattering (CARS) microscopy in tissue | *Arenicola marina*  Marine infaunal lugworm | 10-day acute toxicity test  (OECD/ASTM 1990)  Concentrations 0.003-0.03g/kg | Burrowing behaviour NOEC 0.03g/kg  DNA damage in coelomocytes NOEC 0.03g/kg | [17] |
| SWCNT and MWCNT  (not commercially available)  14C labelled  Dispersed by sonication, mixed to natural sediment. | TEM  TGA  Raman 785 nm  Biological oxidation + (Radioactive labeling) + scintillation counting.  After:  Spectrophotometric | *Lumbriculus variegates*  Infaunal lugworm | Bioaccumulation and elimination  SWCNT 0.003 or 0.03 g/kg d.w.  MWCNT 0.037 or 0.37 g/kg d.w.  Exposure 28 days | Number of worms did not change. | [15] |
| SWCNT  (Shenzhen Nanotech, China)  90%pure  2nm x 5-15µm  >400m^2^/g  MWCNT  (Helix Material Solutions)  >95% pure  MWCNT  (Shenzhen Nanotech, China)  >95% pure  10-20nm x 5-15 µm  >40-400m^2^/g  Nonsonicated/Sonicated  Pure/acid purifyed | EM  SEM  EDS | *Hyalella Azteca*  Amphipod  *Cironomus dilutus*  midge  *Lubriculus variegatus*  oligochaete *Villosa iris*  mussel | 14-d water-only exposures  Nominal concentration 1 g/l | Acid washing removed metal content from the CNT surface and reduced the toxicity compared to pristine CNT, sonication effect was less clear and dependent on species | [59] |
| SWCNT  90% pure (Ni & Co impurities)  (Sigma-Aldrich, St. Louis, MO, USA)  11 nm x 0.5-100 µm  Dispersed by 30 min. Stirring in filtered tap water.  DWCNT  (not commercially available)  98% pure | EDX, but only the amount of C  Inverted microscopy  SEM | *Danio rerio*  zebrafish embryos | 1) SWCNT exposure from 4-96 hpf  Nominal concentrations 20, 40, 60, 120, 240, 360 mg/L  2) DWCNT exposure from 4-96 hpf  Nominal concentrations 240, 360 mg/L | 1) Hatching delay LOEC 120 mg/L.  No effect on embryo development LOEC>360 mg/L.  2) Hatching delay LOEC 240 mg/L. | [19] |
| MWCNT  (Nanostructured and Amorphous Materials)  Carboxylised and FITC-BSA-conjugated by amidation.  20nm x 800nm | TGA  Radioactive labelling  S-TEM  SEM  TEM  AFM  After:  TEM  Inverted microscopy | *Danio rerio*  zebrafish | Full life-cycle assay  Microinjection at 1-cell stage embryos  Concentration 2ng/embryo | Normal development including second generation.  Reduced survival in second generation. | [20] |
| MWCNT  (Nanostructured and Amorphous Materials)  98% pure  Carboxylised and FITC-BSA-conjugated by amidation.  Fluorescent-labelled.  1) Sonicated 48-h  20nm x 200nm  2) Sonicated 24-h  20nm x 800nm | TGA  Radioactive labelling  S-TEM  SEM  TEM  AFM  After:  TEM  Inverted microscopy | *Danio rerio*  zebrafish embryos | Microinjection at 1-cell stage embryos  Concentration 2ng/embryo | 1) severe developmental toxicity  2) NOEC 2ng/embryo | [60] |

| **Particle** | **Characterization**  **method used** | **Model organism** | **Exposure condition, duration**  **and detection method** | **Conclusions** | **Ref** |
| --- | --- | --- | --- | --- | --- |
| MWCNTs  (Shenzhen Nanotech, China)  260nm x 30-40 µm  Stirred in zebrafish medium | TEM  UV-spectroscopy | *Danio rerio*  zebrafish | Fish early life toxicity test (72hpf)  OECD 210  Concentrations 2.5-300mg/L  Fish short-term toxicity test on embryo and sac fry stages  OECD 212  Concentration 5ng (microinjection) | NOEC 40mg/L  LOEC 60mg/L (phenotypic defects)  LOEC 100mg/L (mortality) | [61] |
| DWCNT  (non-commercially available)  1) Sonicated, suspended in artificial seawater.  2) Stirred, suspended in artificial seawater. | Elemental analysis  TEM | *Oryzias melastigma*  medaka fish embryos | Chronic toxicity test  14-d exposure from 2dph.  Concentrations 0, 10, 50, 100mg/L | 1) Growth inhibition LOEC 10mg/L.  2) Growth inhibition LOEC 100mg/L. | [10] |
| SWCNT  (Cheap tubes Inc.)  Stock solutions were dispersed in solvent, sonicated and added to fresh water. | Manufacturer data  TEM  Sandsynligvis ICP-MS | *Oncorhynchus mykiss*  juvenile rainbow trout | 10-day exposure under semi-static conditions  Nominal concentrations 0.1, 0.25 and 0.5mg/L | Absence of oxidative stress or hematological changes. Exposure caused respiratory toxicity, neurotoxicity and hepatotoxicity. | [62] |
| SWCNT  96.3% pure  (Cheep Tubes Inc., Vermont, USA)  1.1 nm x 5-30 µm  Dispersed in Millipore water + 2% SDS  Sonicated for 2.5 h  Steared for 3.5-5.5 h  Sprayed on food  Formed in pellets | Manufacturer data  TEM  Sandsynligvis ICP-MS | *Oncorhynchus mykiss*  juvenile rainbow trout | Dietary toxicity 500mg/kg 2x day for 6 wk and 2 wk recovery | No weight changes, haematological changes, metal accumulation, oxidative tissue injury or gut pathology.  TBARS elevation in brain at 4 wk in exposure. | [63] |

| **Particle** | **Characterization**  **method used** | **Model organism** | **Exposure condition, duration**  **and detection method** | **Conclusions** | **Ref** |
| --- | --- | --- | --- | --- | --- |
| DWCNT  97.7% pure with Co contaminatns  (non-commercially available)  BET 800, 900 m^2^/g  0.7-2.2 nm  Dispersed in reconstituted water.  Ia) without aeration  Ib) with aeration  IIa) without GA  IIb) with GA | 2008:  Element analysis  BET  Raman  SEM  TEM  XRD  After:  Microscope  TEM  Raman  2009:  Element analysis  BET  Raman  SEM  TEM  XRD  After:  Raman | *Xenopus laevis*  african clawed frog larvae | 12-day exposure from developmental stage 50  ISO 21427-1:2006  Micronucleus test (genotoxicity)  I) Nominal concentrations 10, 100, 500mg/L  II) Nominal concentrations 0.1, 1, 10, 50 or 100mg/L | I)  a) Mortality LOEC 500mg/L (85%)  Growth retardation LOEC 10mg/L  b) Mortality LOEC 10mg/L (15%)  Growth retardation LOEC 100mg/L  a + b) Genotoxicity NOEC 500mg/L  II)  a) Mortality and growth retardation LOEC 10mg/L.  b) Growth retardation LOEC 10mg/L  a) Genotoxicity NOEC 100mg/L.  b) Genotoxicity at 1 mg/L. | [22, 23] |
| MWCNT  (Graphistrength C100, Arkema, FR)  97.7% (+Fe)  Dispersed in reconstituted water. | TEM  BET  Raman  After:  Raman 488 nm | *Xenopus laevis*  african clawed frog larvae | 12-day exposure from developmental stage 50  ISO 21427-1:2006  Micronucleus test (genotoxicity)  Nominal concentrations 0.1, 1, 10, 50mg/L | Mortality NOEC 50 mg/L.  Growth retardation LOEC 50mg/L.  Genotoxicity NOEC 50mg/L. | [21] |

| **Particle** | **Characterization**  **method used** | **Model organism** | **Exposure condition, duration**  **and detection method** | **Conclusions** | **Ref** |
| --- | --- | --- | --- | --- | --- |
| MWCNT  (Graphistrength, Arkema, FR)  95% pure (+Al, Fe)  270 m^2^/g  2-20nm x 1-10 µm  Dispersed in reconstituted water.  1) Raw (sonicated)  2) Raw + CMC (0.005%) (rotated)  3) Raw + GA (0.005%) (rotated) | TEM  BET  Raman  After:  Raman 488 nm | *Xenopus laevis*  african clawed frog larvae | 12-day exposure from developmental stage 50  ISO 21427-1:2006  Micronucleus test (genotoxicity)  Nominal concentrations 0.1, 1, 10, 50mg/L  Actual concentrations 0.25, 1.5, 10.1, 50mg/L | Mortality  1+3) LOEC 50 mg/L.  2) LOEC 1 mg/L  Growth retardation  2+3) LOEC 10mg/L.  Growth stimulation  3) 0.1 and 1 mg/L  Genotoxicity  2) 1 and 10mg/L. | [24] |

**TERRESTRIAL TOXICITY**

| **Particle** | **Characterization**  **method used** | **Model organism** | **Exposure condition, duration**  **and detection method** | **Conclusions** | **Ref** |
| --- | --- | --- | --- | --- | --- |
| DWCNT  (SES research)  99.5% pure  <0.4% metals  Surface area 1,255,637nm^2^  10-30nm x 5-15µm  Suspended in DI water, steered in ultrasonic bath, added to dry food. | Manufacturer data | *Eisenia veneta*  soil-dwelling earthworm | 28-day sub-lethal toxicity assay  Concentrations 0, 50, 100, 300, 495mg/ kg dry food  10g of dry food was spiked directly to soil every 7 days | Mortality, growth and hatchability  EC(10) 100mg/kg, EC(50) >495mg/kg  Reproduction effects (cocoon production) EC(10) 37mg/kg, EC(50) 175mg/kg | [64] |
| SWCNT  MWCNT  (Not commercially available)  14C labelled  Sonicated in water for 30 min. and tumbled in soil over night | TEM  TGA  Raman 785 nm | *Eisenia fetida*  Soil-dwelling earthworm | Uptake and Depuration experiments  Nominal concentration  SWCNT 30mg/kg soil d.w.  MWCNT 30 and 300mg/kg soil d.w.  Exposure 28 days | No mortality was observed. | [12] |
| SWCNT  1-2 nm  MWCNT  30-70 nm  (Not commercially available)  Mixed in pyrene spiked soil | TEM  TGA  Raman | *Eisenia fetida*  Soil-dwelling earthworm | Bioaccumulation and elimination  300, 3000mg/kg soil  Exposure 28 days | No mortality was observed. | [13] |
| MWCNT  (Not commercially available)  14C labeled  Non-modified and Modified with PEI with +, - , 0 charge  Reconstitued in DI water, sonicated 30 min. | TEM  NMR (1H)  SEM  Biological oxidation + radioactivity  TGA  Zetasizer | *Eisenia fetida*  Soil-dwelling earthworm | Bioaccumulation and elimination  500mg/kg soil  Exposure 28 days | No mortality observed. | [14] |

| **Particle** | **Characterization**  **method used** | **Model organism** | **Exposure condition, duration**  **and detection method** | **Conclusions** | **Ref** |
| --- | --- | --- | --- | --- | --- |
| SWCNT  70% pure  (Ni:Ycatalyst, CSI, Riverside, CA)  MWCNT (MER, Tuscon, AZ)  Sonicated in solvent. | Manufacturer data  TEM  FE-SEM (field emission SEM) | *Drosophila melanogaster*  fruit fly | Toxicity test (egg to adult)  1) CNT stirred in the larval food, gells with dose 100 and 1000mg/kg  2) CNT dry powder | 1) No effect on egg hatching, general survival.  2) CNT could adhere to fly surfaces and overwhelm natural grooming, impair locomotor function and induce mortality. | [26] |
| SWCNT  (HiPco)  1 nm x 1600 nm  Suspension in Bovine serum albumin was sonicated, centrifuged, decanted and spiked in dry yeast with. | NIR | *Drosophila melanogaster*  fruit fly | Toxicity test (egg to adult)  Concentration 9 mg/L in food | No effect on feeding or survival, despite transfer in body compartments. | [65] |
| SWCNT  1-2nm x 5-30µm (declared)  46.5 nm in DI  115nm in tragacanth gum  90% pure  Hydroxylated  Sonicated in vehicle. | TEM  TRDLS | *Drosophila melanogaster*  fruit fly | Fecundity and fertility assessment  Concentrations 0, 0.005, 0.01, 0.5, 0.1, 0.5% w/v in DI | NOEC 0.5% w/v | [66] |
|  | TEM  TRDLS | CD-1 mouse | Fetal terratogenicity (GD 19)  Oral exposure GD 9 to 0, 10, 100mg/kg in 0.5% w/w tragacanth gum | No effect litter size, weight,.  0.01g/kg induced resorptions, gross morphological defects, skeletal abnormalities.  0.1 g/kg no effect. |  |
| SWCNT  (Thomas Swan and Co Ltd, UK)  98%pure  2% Fe, ‰Co, Ni, Mn  0.9–1.7 nm x < 1 μm  Suspended in saline or corn oil, sonicated excesively | DLS | *Fisher rat* | Toxicity test  Oral gavage  Concentration 0.064or 0.64mg/kg | Genotoxicity lung & liver  LOEC 0.064 mg/kg  (8-oxo-7,8-dihydro-2'-deoxyguanosine levels) | [67] |

| **Particle** | **Characterization**  **method used** | **Model organism** | **Exposure condition, duration**  **and detection method** | **Conclusions** | **Ref** |
| --- | --- | --- | --- | --- | --- |
| MWCNT CM-95  (Incheon, Korea)  15-15nm x 20 μm  95% pure  5% Fe | Manufacturer data | *Sprague-Dawley rat* | Reproductive toxicity  Oral gavage on gestation day 6-19  Concentrations 0, 8, 40, 200, 1000mg/kg/day | NOEC 1g/kg/day reproductive toxicity  LOEC 1g/kg/day maternal immune function (thymus weight) | [68] |
| SWCNT  (Carbon Solutions, Riverside, CA)  P3-SWCNT non-functionalyzed  80-90% pure  5-10% metal content by wt  P8-SWCNT functionalyzed  80-90% pure  3% metal content by wt  Dispersed in DI water and sonicated for 30 min and 90 min | SEM  After:  SEM | USEPA 850.4150 Tier I Guideline plants:  *Brassica oleracea*  Cabage  *Daucus carota*  Carrot  *Cucumis sativus*  Cucumber  *Allium cepa*  Onion  *Lycopersicon Esculentum*  Tomato  *Luctuca sativa*  Lettuce | Seedling exposure for 24 and 48 h  (Root elongation test)  Final concentrations P8-SWCNT 9, 56, 104, 315, 1750mg/L  Final concentrations P3-SWCNT 56, 315, 1750mg/L | P3-SWCNT  Inhibited root elongation in tomato (24 & 48 h).  Enhanced root elongation in onion and cucumber (24 h).  P8-SWCNT  Inhibited root elongation in lettuce (48 h, exp.1; no effect exp. 2).  P3>P8  24 h>48 h | [30] |
| MWCNT  (not comertially available)  10-35nm x 6 µm  98.5% pure  carboxylized  SWCNT  (not comertially available)  1-2 nm x ~2 µm  98% pure  Carboxylized  Dispersed in Marushige and Skoog growth medium. | PT  PA  Optical microscope Raman  TEM | Tomato plant  *Solanum lycopersicum* | Plant growth  Nominal concentrations 50 mg/L | Both CNT enhanced plant growth.  Microarray analyses indicated that gene expression was altered for stress related genes and water channel genes. | [32] |

| **Particle** | **Characterization**  **method used** | **Model organism** | **Exposure condition, duration**  **and detection method** | **Conclusions** | **Ref** |
| --- | --- | --- | --- | --- | --- |
| MWCNT  (not commercially available)  98% pure  Dispersed in Marushige and Skoog growth medium. | TEM  TGA  Raman | Tomato plant  *Solanum lycopersicum* | Germination rate  Nominal concentrations 10. 20, 40 mg/L | All concentrations CNT enhanced germination and biomass production. | [33] |
| MWCNT  (Arry International, GE)  60% pure  ~30 nm  O-MWCNT (oxidized)  ~20 nm  Dispersed in DI and sonicated | FTIR  FESEM  XRD  After:  TGA  Conductivity  UV/VIS | Mustard plant  *Brassica* | Time-50 Germination  Nominal concentration  MWCNT 23, 46 mg/L  O-MWCNT 2.3, 6.9 mg/L | MWCNT and O-MWCNT  23 and 2.3 mg/L ↑ biomass, root elongation, germination index (GI)  46 and 6.9 mg/L ↓ biomass, root elongation, GI  O-MWCNT seed pre-treatment reduced time-50 germination by 44% compared to water pre-treatment. | [31] |
| MWCNT  (Shenzhen nanotech Port Co., LTD)  >95%pure  10-20nm x 1-2 µm  Actual 126m^2^/g  Dispersed in DI water in UV-bath for 30min. | BET  AFM | *Brassica napus*  Rape  *Raphanus sativus*  Radish  *Lolium perenne*  Ryegrass  *Lactuca sativa*  Lettuce  *Zea mays*  Corn  *Cucumis sativus*  Cucumber | Seed germination and root growth  (OPPTS 850.4200, USA EPA)  Soaked for 2-h and 5 day follow up.  Concentration 2000mg/L | NOEC 2000mg/L germination and seed growth. | [69] |

| **Particle** | **Characterization**  **method used** | **Model organism** | **Exposure condition, duration**  **and detection method** | **Conclusions** | **Ref** |
| --- | --- | --- | --- | --- | --- |
| MWCNT  (not commercially available)  Dispersed in 25% Hoagland solution and sonicated. | Manufacturer data | *Cucurbita pepo*  zucchini plant | 15-day growth test  (germination and hydroponic biomass)  Concentration 1000mg/L in 25% Hoagland solution | No effect on germination and root elongation.  Reduced growth by 60%. | [70] |
| MWCNT  (Sigma-Aldrich)  110-170nm x 9µm  Excitation 710nm  Emission 360-530nm  Sonicated for 12h in suspension | TPEM  (two-photon excitation  Microscopy) | Wheat  *Triticum* | Root growth 96-h intervals  Nominal concentrations 100 mg/L | No effect on root, shoot or leaf growth.. | [34] |
| MWCNT  >95% pure  (Shenzhen Nanotech Co., China)  MWCNT10  9.4 nm (TEM)  357 m^2^/g (BET)  MWCNT60  42.7 nm (TEM)  73 m^2^/g (BET)  Sewage sludge were spiked with CNTs mixed and sludge was mixed in soil ratio 1:19 (w/w) | TEM  BET  Element analysis | *Leppidium sativum*  Cress  *Sorghum saccharatum*  Sorgo  *Solanum lycopersicon*  Tomato  *Raphanus sativus*  Radish  *Cucumis sativus*  Cucumber | Phytotoxkit microbiotest (3-day)  In OECD soil 1984  4 sludge types  Concentrations in sludge 0.01, 0.1, 0.5% (w/w) | Phytotoxicity dependent on NT diameter, type sewage on plant tested.  Phytotoxicity NOEC>0.5%(w/w) | [71] |

| **Particle** | **Characterization**  **method used** | **Model organism** | **Exposure condition, duration**  **and detection method** | **Conclusions** | **Ref** |
| --- | --- | --- | --- | --- | --- |
| MWCNT  90% pure  (Sigma-Aldrich)  Density 2.1 g/cm^3^ at 25°C  10-15 nm x 0.1-10 µm  2.3% Al, 1.9% Fe  Dispered by mixing for 30 min + sonication for 1h + mixing for 48h + sonication for 10 min | Manufacturer data  SEM  Element analysis PIXE | Fresh activated sludge with and without extracellular polymeric substances (EPS)  a) Sheared (with EPS)  b) Un-sheared (without EPS) | Activated sludge respiration inhibition test  Final concentrations 0.64, 1.44, 2.16, 3.24g/L | Effect dose 1.44 and 2.16 g/L in both types sludge.  MWCNT toxicity Sheared > Un-Sheared  (EPS has protective function, free EPS in sheared solutions disaggregated MCWNT ↑ toxicity) | [72] |

D: hydrodynamic diameter of aggregates, Z: zeta potential, BSA: bovine serum albumin, FE-SEM (field emission SEM) LPC: Lysophophatidylchlorine, PEI: polyethyleneimine, CMC: either carboxymethylcellulose, GA: gum arabic

References

1 Ghafari P, St-Denis CH, Power ME, Jin X, Tsou V, Mandal HS et al. Impact of carbon nanotubes on the ingestion and digestion of bacteria by ciliated protozoa. Nat Nanotechnol 2008;3**:**347-351.

2 Zhu Y, Zhao Q, Li Y, Cai X, Li W. The interaction and toxicity of multi-walled carbon nanotubes with Stylonychia mytilus. J Nanosci Nanotechnol 2006;6**:**1357-1364.

3 Zhu X., Zhu L., Chen Y., Tian S. Acute toxicities of six manufactured nanomaterial suspensions to Daphnia manga. J Nanopart Res 2009;11**:**67-75.

4 Edgington AJ, Roberts AP, Taylor LM, Alloy MM, Reppert J, Rao AM et al. The influence of natural organic matter on the toxicity of multiwalled carbon nanotubes. Environ Toxicol Chem 2010;29**:**2511-2518.

5 Roberts AP, Mount AS, Seda B, Souther J, Qiao R, Lin S et al. In vivo biomodification of lipid-coated carbon nanotubes by Daphnia magna. Environ Sci Technol 2007;41**:**3025-3029.

6 Petersen EJ, Akkanen J, Kukkonen J, Weber WJ, Jr. Biological uptake and depuration of carbon nanotubes by *Daphnia magna*. Environ Sci Technol 2009;43**:**2969-2975.

7 Petersen EJ, Pinto RA, Mai DJ, Landrum PF, Weber WJ, Jr. Influence of polyethyleneimine graftings of multi-walled carbon nanotubes on their accumulation and elimination by and toxicity to Daphnia magna. Environ Sci Technol 2011;45**:**1133-1138.

8 Li M, Huang CP. The responses of Ceriodaphnia dubia toward multi-walled carbon nanotubes: Effect of physical-chemical treatment. Carbon 2011;49**:**1672-1679.

9 Kennedy AJ, Hull MS, Steevens JA, Dontsova KM, Chappell MA, Gunter JC et al. Factors influencing the partitioning and toxicity of nanotubes in the aquatic environment. Environ Toxicol Chem 2008;27**:**1932-1941.

10 Kwok KW, Leung KM, Flahaut E, Cheng J, Cheng SH. Chronic toxicity of double-walled carbon nanotubes to three marine organisms: influence of different dispersion methods. Nanomedicine (Lond) 2010;5**:**951-961.

11 Templeton RC, Ferguson PL, Washburn KM, Scrivens WA, Chandler GT. Life-cycle effects of single-walled carbon nanotubes (SWNTs) on an estuarine meiobenthic copepod. Environmental Science & Technology 2006;40**:**7387-7393.

12 Petersen EJ, Huang QG, Weber WJ. Bioaccumulation of radio-labeled carbon nanotubes by *Eisenia foetida*. Environ Sci Technol 2008;42**:**3090

13 Petersen EJ, Pinto RA, Landrum PF, Weber J. Influence of Carbon Nanotubes on Pyrene Bioaccumulation from Contaminated Soils by Earthworms. Environmental Science & Technology 2009;43**:**4181-4187.

14 Petersen EJ, Pinto RA, Zhang L, Huang Q, Landrum PF, Weber WJ. Effects of polyethyleneimine-mediated functionalization of multi-walled carbon nanotubes on earthworm bioaccumulation and sorption by soils. Environ Sci Technol 2011;45**:**3718-3724.

15 Petersen EJ, Huang QG, Weber WJ. Ecological uptake and depuration of carbon nanotubes by Lumbriculus variegatus. Environmental Health Perspectives 2008;116**:**496-500.

16 Petersen EJ, Huang Q, Weber WJ, Jr. Relevance of octanol-water distribution measurements to the potential ecological uptake of multi-walled carbon nanotubes. Environ Toxicol Chem 2010;29**:**1106-1112.

17 Galloway T, Lewis C, Dolciotti I, Johnston BD, Moger J, Regoli F. Sublethal toxicity of nano-titanium dioxide and carbon nanotubes in a sediment dwelling marine polychaete. Environmental Pollution 2010;158**:**1748-1755.

18 Ferguson PL, Chandler GT, Templeton RC, Demarco A, Scrivens WA, Englehart BA. Influence of sediment-amendment with single-walled carbon nanotubes and diesel shoot on bioaccumulation of hydrophobic organic contaminats by bentic invertebrates. Environ Sci Technol 2008;42**:**3879

19 Cheng J, Flahaut E, Cheng SH. Effect of carbon nanotubes on developing zebrafish (Danio rerio) embryos. Environ Toxicol Chem 2007;26**:**708-716.

20 Cheng JP, Chan CM, Veca LM, Poon WL, Chan PK, Qu LW et al. Acute and long-term effects after single loading of functionalized multi-walled carbon nanotubes into zebrafish (Danio rerio). Toxicology and Applied Pharmacology 2009;235**:**216-225.

21 Mouchet F, Landois P, Puech P, Pinelli E, Flahaut E, Gauthier L. Carbon nanotube ecotoxicity in amphibians: assessment of multiwalled carbon nanotubes and comparison with double-walled carbon nanotubes. Nanomedicine 2010;5**:**963-974.

22 Mouchet F, Landois P, Sarremejean E, Bernard G, Puech P, Pinelli E et al. Characterisation and in vivo ecotoxicity evaluation of double-wall carbon nanotubes in larvae of the amphibian Xenopus laevis. Aquatic Toxicology 2008;87**:**127-137.

23 Mouchet F, Landois P, Datsyuk V, Puech P, Pinelli E, Flahaut E et al. International amphibian micronucleus standardized procedure (ISO 21427-1) for in vivo evaluation of double-walled carbon nanotubes toxicity and genotoxicity in water. Environ Toxicol 2009;

24 Bourdiol F, Mouchet F, Perrault A, Fourquaux I, Datas L, Gancet C et al. Biocompatible polymer-assisted dispersion of multi walled carbon nanotubes in water, application to the investigation of their ecotoxicity using Xenopus laevis amphibian larvae. Carbon 2013;54**:**175-191.

25 Leeuw TK, Reith RM, Simonette RA, Harden ME, Cherukuri P, Tsyboulski DA et al. Nano Lett 2007;7**:**2650

26 Liu XY, Vinson D, Abt D, Hurt RH, Rand DM. Differential Toxicity of Carbon Nanomaterials in Drosophila: Larval Dietary Uptake Is Benign, but Adult Exposure Causes Locomotor Impairment and Mortality. Environmental Science & Technology 2009;43**:**6357-6363.

27 Wang H, Wang J, Deng X, Sun H, Shi Z, Gu Z et al. Biodistribution of carbon single-wall carbon nanotubes in mice. J Nanosci Nanotechnol 2004;4**:**1019-1024.

28 Deng X, Jia G, Wang H, Sun H, Wang X, Yang S et al. Translocation and fate of multi-walled carbon nanotubes in vivo. Carbon 2007;45**:**1419-1424.

29 Lin S, Reppert J, Hu Q, Hudson JS, Reid ML, Ratnikova TA et al. Uptake, Translocation, and Transmission of Carbon Nanomaterials in Rice Plants. Small 2009;5**:**1128-1132.

30 Canas JE, Long M, Nations S, Vadan R, Dai L, Luo M et al. Effects of functionalized and nonfunctionalized single-walled carbon nanotubes on root elongation of select crop species. Environ Toxicol Chem 2008;27**:**1922-1931.

31 Mondal A, Basu R, Das S, Nandy P. Beneficial role of carbon nanotubes on mustard plant growth: an agricultural prospect. Journal of Nanoparticle Research 2011;13**:**4519-4528.

32 Khodakovskaya MV, de Silva K, Nedosekin DA, Dervishi E, Biris AS, Shashkov EV et al. Complex genetic, photothermal, and photoacoustic analysis of nanoparticle-plant interactions. Proceedings of the National Academy of Sciences of the United States of America 2011;108**:**1028-1033.

33 Khodakovskaya M, Dervishi E, Mahmood M, Xu Y, Li ZR, Watanabe F et al. Carbon Nanotubes Are Able To Penetrate Plant Seed Coat and Dramatically Affect Seed Germination and Plant Growth. ACS Nano 2009;3**:**3221-3227.

34 Wild E, Jones KC. Novel Method for the Direct Visualization of in Vivo Nanomaterials and Chemical Interactions in Plants. Environmental Science & Technology 2009;43**:**5290-5294.

35 Kang S, Pinault M, Pfefferle LD, Elimelech M. Single-walled carbon nanotubes exhibit strong antimicrobial activity. Langmuir 2007;23**:**8670-8673.

36 Rodrigues DF, Elimelech M. Toxic Effects of Single-Walled Carbon Nanotubes in the Development of E. coli Biofilm. Environmental Science & Technology 2010;44**:**4583-4589.

37 Kang S, Herzberg M, Rodrigues DF, Elimelech M. Antibacterial effects of carbon nanotubes: Size does matter! Langmuir 2008;24**:**6409-6413.

38 Kang S, Mauter MS, Elimelech M. Physicochemical determinants of multiwalled carbon nanotube bacterial cytotoxicity. Environ Sci Technol 2008;42**:**7528-7534.

39 Liu S, Wei L, Hao L, Fang N, Chang MW, Xu R et al. Sharper and Faster Nano Darts Kill More Bacteria: A Study of Antibacterial Activity of Individually Dispersed Pristine Single-Walled Carbon Nanotube. ACS Nano 2009;3**:**3891-3902.

40 Arias LR, Yang L. Inactivation of bacterial pathogens by carbon nanotubes in suspensions. Langmuir 2009;25**:**3003-3012.

41 Yang C, Mamouni J, Tang Y, Yang L. Antimicrobial activity of sigle-walled carbon nanotubes: Lenght effect. Lugmuir 2010;26**:**16013-16019.

42 Vecitis CD, Zodrow KR, Kang S, Elimelech M. Electronic-structure-dependent bacterial cytotoxicity of single-walled carbon nanotubes. ACS Nano 2010;4**:**5471-5479.

43 Kang S, Mauter MS, Elimelech M. Microbial cytotoxicity of carbon-based nanomaterials: implications for river water and wastewater effluent. Environ Sci Technol 2009;43**:**2648-2653.

44 Zheng H, Liu L, Lu Y, Long Y, Wang L, Ho KP et al. Rapid determination of nanotoxicity using luminous bacteria. Anal Sci 2010;26**:**125-128.

45 Jin L, Son Y, Yoon TK, Kang YJ, Kim W, Chung H. High concentrations of single-walled carbon nanotubes lower soil enzyme activity and microbial biomass. Ecotoxicol Environ Saf 2013;88**:**9-15.

46 Riding MJ, Martin FL, Trevisan J, Llabjani V, Patel II, Jones KC et al. Concentration-dependent effects of carbon nanoparticles in gram-negative bacteria determined by infrared spectroscopy with multivariate analysis. Environmental pollution (Barking, Essex : 1987) 2012;163

47 Simon-Deckers A, Loo S, Mayne-L'hermite M, Herlin-Boime N, Menguy N, Reynaud C et al. Size-, Composition- and Shape-Dependent Toxicological Impact of Metal Oxide Nanoparticles and Carbon Nanotubes toward Bacteria. Environmental Science & Technology 2009;43**:**8423-8429.

48 Chung H, Son Y, Yoon TK, Kim S, Kim W. The effect of multi-walled carbon nanotubes on soil microbial activity. Ecotoxicology and Environmental Safety 2011;74**:**569-575.

49 Velzeboer I, Kupryianchyk D, Peeters ETHM, Koelmans AA. Community effects of carbon nanotubes in aquatic sediments. Environment International 2011;37**:**1126-1130.

50 Blaise C, Gagne F, Ferard JF, Eullaffroy P. Ecotoxicity of selected nano-materials to aquatic organisms. Environ Toxicol 2008;23**:**591-598.

51 Youn S, Wang R, Gao J, Hovespyan A, Ziegler KJ, Bonzongo JC et al. Mitigation of the impact of single-walled carbon nanotubes on a freshwater green algae: Pseudokirchneriella subcapitata. Nanotoxicology 2011;

52 Schwab F, Bucheli TD, Lukhele LP, Magrez A, Nowack B, Sigg L et al. Are carbon nanotube effects on green algae caused by shading and agglomeration? Environ Sci Technol 2011;45**:**6136-6144.

53 Long Z, Ji J, Yang K, Lin D, Wu F. Systematic and quantitative investigation of the mechanism of carbon nanotubes' toxicity toward algae. Environ Sci Technol 2012;46**:**8458-8466.

54 Wei LP, Thakkar M, Chen YH, Ntim SA, Mitra S, Zhang XY. Cytotoxicity effects of water dispersible oxidized multiwalled carbon nanotubes on marine alga, Dunaliella tertiolecta. Aquatic Toxicology 2010;100**:**194-201.

55 Kim KT, Klaine SJ, Lin S, Ke PC, Kim SD. Acute toxicity of a mixture of copper and single-walled carbon nanotubes to Daphnia magna. Environ Toxicol Chem 2010;29**:**122-126.

56 Alloy MM, Roberts AP. Effects of suspended multi-walled carbon nanotubes on daphnid growth and reproduction. Ecotoxicol Environ Saf 2011;74**:**1839-1843.

57 Kim KT, Edgington AJ, Klaine SJ, Cho JW, Kim SD. Influence of multiwalled carbon nanotubes dispersed in natural organic matter on speciation and bioavailability of copper. Environ Sci Technol 2009;43**:**8979-8984.

58 Kennedy AJ, Gunter JC, Chappell MA, Goss JD, Hull MS, Kirgan RA et al. Influence of nanotube preparation in aquatic bioassays. Environ Toxicol Chem 2009;28**:**1930-1938.

59 Mwangi JN, Wang N, Ingersoll CG, Hardesty DK, Brunson EL, Li H et al. Toxicity of carbon nanotubes to freshwater aquatic invertebrates. Environ Toxicol Chem 2012;31**:**1823-1830.

60 Cheng J, Cheng SH. Influence of carbon nanotube length on toxicity to zebrafish embryos. Int J Nanomedicine 2012;7**:**3731-3739.

61 Asharani PV, Serina NG, Nurmawati MH, Wu YL, Gong Z, Valiyaveettil S. Impact of multi-walled carbon nanotubes on aquatic species. J Nanosci Nanotechnol 2008;8**:**3603-3609.

62 Smith CJ, Shaw BJ, Handy RD. Toxicity of single walled carbon nanotubes to rainbow trout, (Oncorhynchus mykiss): Respiratory toxicity, organ pathologies, and other physiological effects. Aquatic Toxicology 2007;82**:**94-109.

63 Fraser TW, Reinardy HC, Shaw BJ, Henry TB, Handy RD. Dietary toxicity of single-walled carbon nanotubes and fullerenes (C(60)) in rainbow trout (Oncorhynchus mykiss). Nanotoxicology 2011;5**:**98-108.

64 Scott-Fordsmand JJ, Krogh PH, Schaefer M, Johansen A. The toxicity testing of double-walled nanotubes-contaminated food to Eisenia veneta earthworms. Ecotoxicology and Environmental Safety 2008;71**:**616-619.

65 Leeuw TK, Reith RM, Simonette RA, Harden ME, Cherukuri P, Tsyboulski DA et al. Nano Lett 2007;7**:**2650

66 Philbrook NA, Walker VK, Afrooz ARMN, Saleh NB, Winn LM. Investigating the effects of functionalized carbon nanotubes on reproduction and development in Drosophila melanogaster and CD-1 mice. Reproductive Toxicology 2011;32**:**442-448.

67 Folkmann JK, Risom L, Jacobsen NR, Wallin H, Loft S, Møller P. Oxidatively damaged DNA in rats exposed by oral gavage to C60 fullerenes and single-walled carbon nanotubes. Environ Health Perspect 2009;117**:**703-708.

68 Lim JH, Kim SH, Lee IC, Moon C, Kim SH, Shin DH et al. Evaluation of Maternal Toxicity in Rats Exposed to Multi-Wall Carbon Nanotubes during Pregnancy. Environ Health Toxicol 2011;26**:**e2011006

69 Lin D, Xing B. Phytotoxicity of nanoparticles: inhibition of seed germination and root growth. Environ Pollut 2007;150**:**243-250.

70 Stampoulis D, Sinha SK, White JC. Assay-Dependent Phytotoxicity of Nanoparticles to Plants. Environmental Science & Technology 2009;43**:**9473-9479.

71 Oleszczuk P, Josko I, Xing B. The toxicity to plants of the sewage sludges containing multiwalled carbon nanotubes. J Hazard Mater 2011;186**:**436-442.

72 Luongo LA, Zhang XJ. Toxicity of carbon nanotubes to the activated sludge process. J Hazard Mater 2010;178**:**356-362.
